# gCAnno: a graph-based single cell type annotation method

**DOI:** 10.1186/s12864-020-07223-4

**Published:** 2020-11-23

**Authors:** Xiaofei Yang, Shenghan Gao, Tingjie Wang, Boyu Yang, Ningxin Dang, Kai Ye

**Affiliations:** 1 School of Computer Science and Technology, Faculty of Electronic and Information Engineering, Xi’an Jiaotong University, Xi’an, Shaanxi China; 2 MOE Key Lab for Intelligent Networks & Networks Security, Faculty of Electronic and Information Engineering, Xi’an Jiaotong University, Xi’an, Shaanxi China; 3 School of Automation Science and Engineering, Faculty of Electronic and Information Engineering, Xi’an Jiaotong University, Xi’an, Shaanxi China; 4 Genome Institute, The First Affiliated Hospital of Xi’an Jiaotong University, Xi’an, Shaanxi China; 5 The School of Life Science and Technology, Xi’an Jiaotong University, Xi’an, Shaanxi China

**Keywords:** Graph embedding, Cell type annotation, Single cell RNA analysis

## Abstract

**Background:**

Current single cell analysis methods annotate cell types at cluster-level rather than ideally at single cell level. Multiple exchangeable clustering methods and many tunable parameters have a substantial impact on the clustering outcome, often leading to incorrect cluster-level annotation or multiple runs of subsequent clustering steps. To address these limitations, methods based on well-annotated reference atlas has been proposed. However, these methods are currently not robust enough to handle datasets with different noise levels or from different platforms.

**Results:**

Here, we present gCAnno, a graph-based Cell type Annotation method. First, gCAnno constructs cell type-gene bipartite graph and adopts graph embedding to obtain cell type specific genes. Then, naïve Bayes (gCAnno-Bayes) and SVM (gCAnno-SVM) classifiers are built for annotation. We compared the performance of gCAnno to other state-of-art methods on multiple single cell datasets, either with various noise levels or from different platforms. The results showed that gCAnno outperforms other state-of-art methods with higher accuracy and robustness.

**Conclusions:**

gCAnno is a robust and accurate cell type annotation tool for single cell RNA analysis. The source code of gCAnno is publicly available at https://github.com/xjtu-omics/gCAnno.

**Supplementary Information:**

The online version contains supplementary material available at 10.1186/s12864-020-07223-4.

## Background

Bulk RNA sequencing measures average gene expression level in a large population of cells, hindering dissection of heterogeneous cell types [1]. In 2009, single cell RNA sequencing (scRNA-seq) technology was developed to provide valuable insights into cell heterogeneity [2].

In general, accurate cell type annotation for single cell data is a prerequisite for any further investigation of cell heterogeneous [3–6]. The commonly used cell type annotation methods, including Seurat [7], SCANPY [8] and SINCERA [9], adopts a similar procedure of data quality control, reads mapping, UMI quantification, expression normalization, clustering, differentially expressed genes (DEGs) of each cluster identification and cell type assignment based on biomarker genes [10]. However, those methods report cluster-level rather than truly single cell-level annotation results, masking subtle differences within each cluster. In addition, different clustering methods and many tunable parameters led to uncertain clustering outcome. These above two factors cause incorrect cluster-level annotations or multiple runs of subsequent clustering steps [10].

To overcome the above issues, two distinct strategies, namely biomarker-based and reference-based approaches, have been proposed. The biomarker-based methods, such as Garnett [11] and CellAssign [12], aim to establish mappings between the query dataset and the well-studied biomarkers. In particular, Garnett trains a classifier based on the user defined markup language. CellAssign builds a probabilistic model that leverages prior knowledge of cell-type marker genes for annotation. However, collecting a comprehensive biomarker set of different cell types is cumbersome, time-consuming and subjective. Thus recently reference-based approaches, such as Scmap [13], Chetah [14] and scPred [15] have been developed and are gaining popularity after a number of well-annotated single cell data were published, especially the datasets released by human cell atlas (HCA) [16]. The reference-based methods follow data-driven strategy and construct mappings between query dataset and the well-annotated reference datasets. For example, Scmap uses drop-based method to select feature genes as variables and constructs mapping by distance and correlation coefficient. Another method, scPred selects differential principle components (PCs) calculated by gene expression value between cell types and trains an SVM model with these PCs. Recently, a comprehensive benchmark study [17] of 22 cell type classification methods indicated that SVM classifier has overall the best performance. However, these methods are sensitive to experiment batches, sequencing platforms and noises, all of which are intrinsic properties of the single cell datasets.

**Fig. 1:**
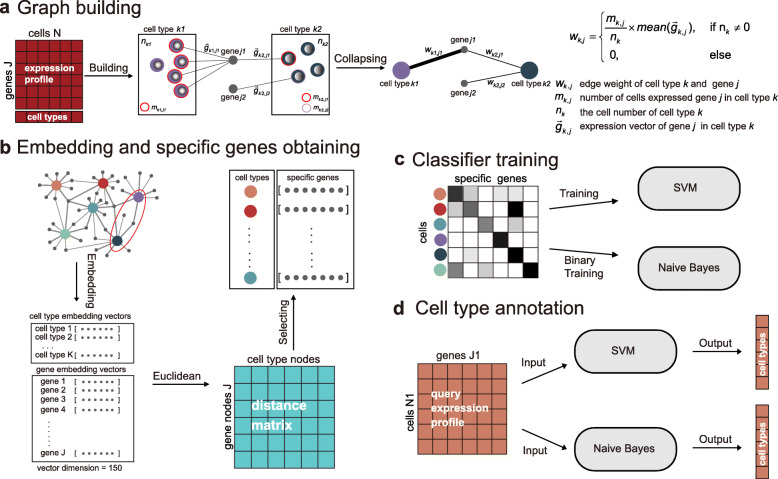
Overview of gCAnno. **a** Cell type-gene graph building. The graph contains gene nodes (gray circles) and cell type nodes (other color circles). **b** Graph embedding converts graphs into low dimensional vectors. Genes are selected based on the distance between the two types of vectors. **c** Training Naïve Bayes and SVM classifiers for annotation. **d** Cell type annotation for new query dataset

Here, we propose a reference-based method, gCAnno, using graph representation feature selection strategy to comprehensively represent the global view of associations between cell types and genes for robust and high accuracy single cell-level annotation. Our gCAnno method starts with construction of a weighted cell type-gene bipartite graph. Then, graph embedding is applied to capture the cell type specific genes and naïve Bayes (gCAnno-Bayes) and SVM (gCAnno-SVM) classifiers are built for further annotation (Fig. 1). We compared gCAnno with the state-of-the-art methods on four published datasets as the basic test [3–6]. We also reported the performance comparison on large dataset with deep annotation level [18], different single cell platforms, simulated datasets with either various cell type imbalance situations and different dropout noise levels as the advanced test. Finally, runtime is summarized to demonstrate the efficiency of gCAnno.

## Results

To evaluate the performance of gCAnno, we first evaluated the cell type-gene specific relation, and then compared gCAnno with five state-of-art methods, including Scmap-cell, Scmap-cluster, Chetah, scPred and SVM, in the following four aspects: 1) cell type specificity of gCAnno detected genes, 2) overall performance on different scRNA-seq datasets, 3) robustness test on simulated drop-out and imbalance noise data, 4) cross platform annotation.

### Cell type specificity of gene sets detected by gCAnno

**Fig. 2:**
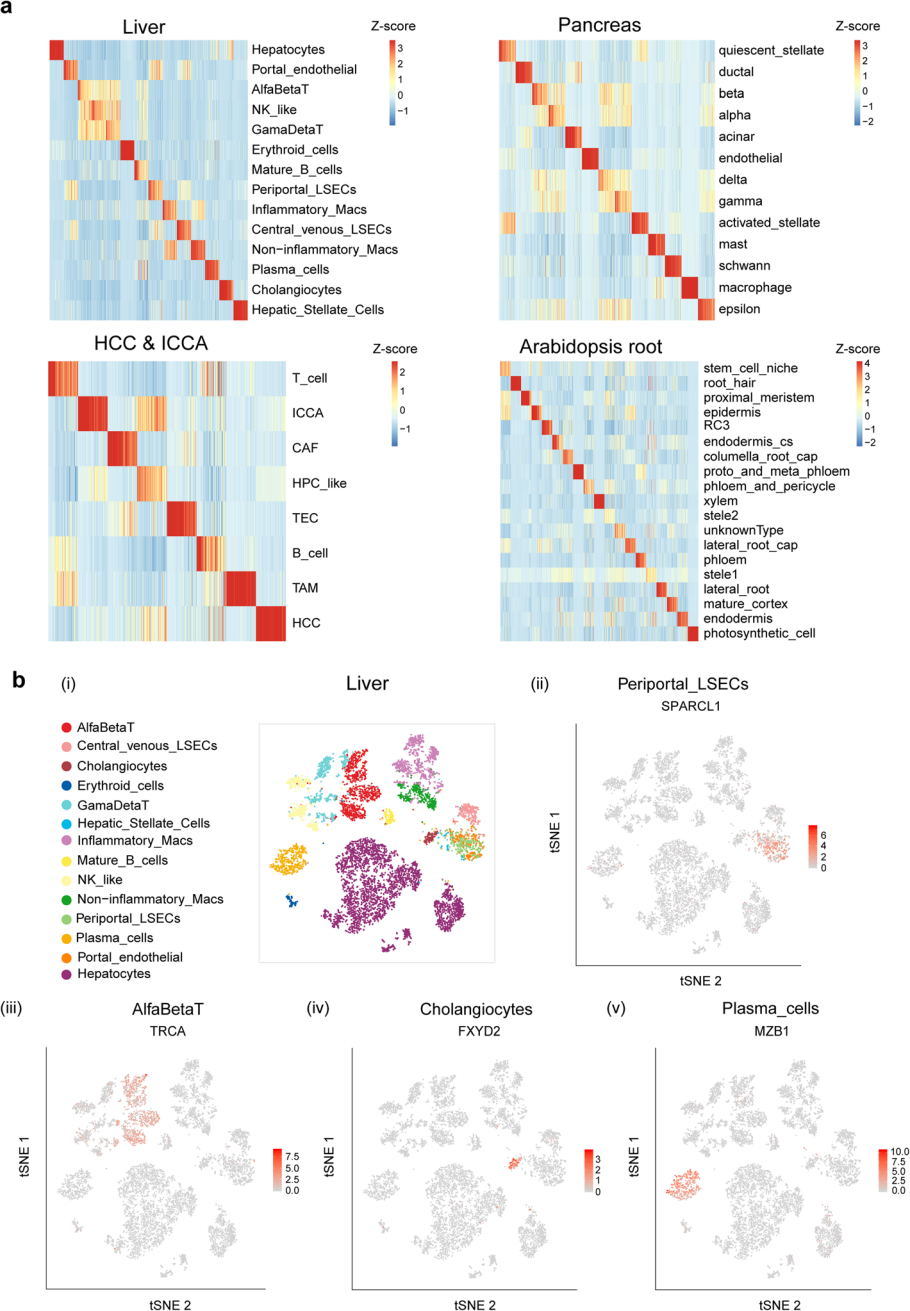
Cell type specificity of gene sets detected by gCAnno. **a** The heatmap of the expression of each cell type specific gene sets in liver, pancreas, HCC & ICCA and AT root datasets. The expression values are normalized by *z*-score across different cell types. **b** t-SNE plots showing the expression of rank one cell specific gene in four cell types in liver dataset. (i) t-SNE projection showing a reference map of all cell types. The expression of (ii) *SPARCL1* in Periportal_LSECs, (iii) *TRCA* in AlfaBetaT, (iv) *FXYD2* in Cholangiocytes and (v) *MZB1* in Plasma_cells (t-SNE plots for all cell types are in Additional file 10: Figure S6)

After graph embedding step, gCAnno selects cell type specific gene sets, which largely determines the performance of our approach. Thus, we first evaluated the cell type specificity of gene sets detected in the four datasets. We noticed that clear cell type specific expression patterns are observed for these selected genes (Fig. 2; Additional file 9: Figure S5; Additional file 10: Figure S6). Among the reported marker genes from the corresponding publications, gCAnno is able to capture an average of 57% of them, indicating gCAnno’s effectiveness of cell type specific gene identification (Additional file 11: Figure S7; Additional file 12: Table S4).

### Overall and large dataset performance evaluation

We next evaluated and compared overall performance of gCAnno, Scmap, scPred, Chetah and SVM with four published scRNA-seq datasets (Table 1). We found that the comprehensive kappa coefficient of both gCAnno was consistently much higher than those of Scmap-cluster, Scmap-cell and scPred, respectively (*p* < 0.05, Wilcoxon rank sum test) (Fig. 3a-d) (Additional file 13: Table S5), hinting gCAnno’s better performance than other methods on cell type annotation across different species (e.g. human or plant), organs (e.g. liver or pancreases), or disease states (e.g. health or cancer). In 20 mouse organs dataset, the comprehensive kappa coefficient of both gCAnno were 0.74 (gCAnno-Bayes) and 0.94 (gCAnno-SVM), and other methods achieve 0.16 (Scmap-cluster), 0.18 (Scmap-cell), 0.80 (Chetah), 0.63 (scPred) and 0.92 (SVM), respectively (Fig. 3e). We found that gCAnno-SVM achieved highest performance than other methods in large dataset with deep annotation level (Additional file 6: Table S2; Additional file 14: Figure S8).

**Table 1:** The list of scRNA-seq datasets in overall performance test

Dataset	#Cells	#Genes	# Cell types
Liver [4]	8444	20,007	14
Pancreas [3]	8562	20,126	13
AT root [6]	7053	32,833	19
HCC, ICCA [5]	4729	19,379	8

# means the number of

**Fig. 3:**
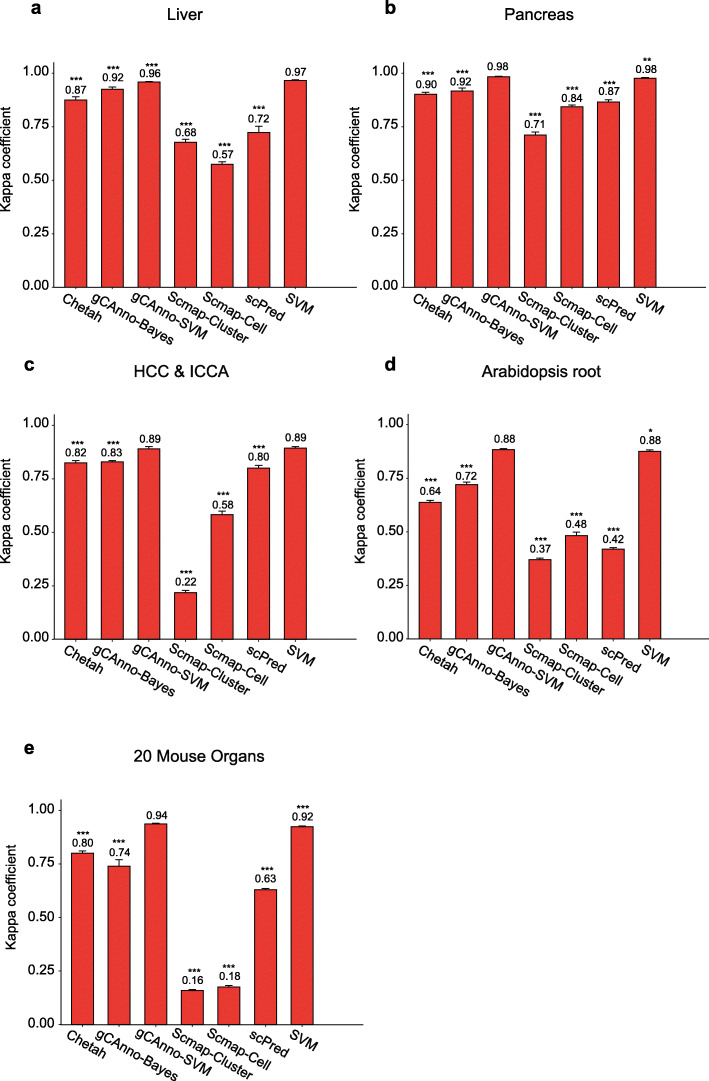
Overall performance evaluation. Comparisons of gCAnno with Scmap-Cluster, Scmap-Cell, scPred, Chetah and SVM based on kappa coefficient on **a** liver dataset, **b** pancreas dataset, **c** HCC & ICCA dataset, and **d** AT root dataset. *: *p*-values < 0.1; **: *p*-values < 0.05; ***: *p*-values < 0.01, Wilcoxon rank sum test. The number is the mean of five cross validation. The error bar is the standard deviation. The *y*-axis is the kappa coefficient

### Robustness on dropout and imbalance noisy data

**Fig. 4:**
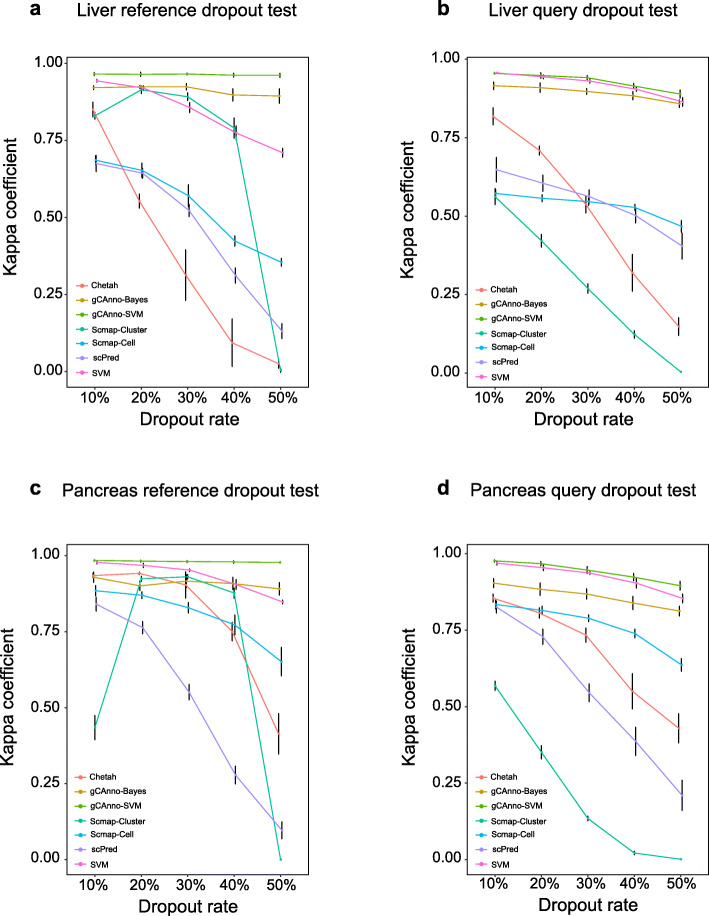
Robustness performance evaluation. Robustness of dropout noise comparisons of gCAnno with Scmap-Cluster, Scmap-Cell, scPred, Chetah and SVM on **a** liver reference dropout dataset, **b** liver query dropout dataset, **c** pancreas reference dropout dataset, **d** pancreas query dropout dataset. The middle point is the mean kappa coefficients of five-fold cross validation. The error bar is the standard deviation. The *y*-axis is the kappa coefficient and the *x*-axis is the dropout rate

Besides basic accuracy, we examined its robustness in the presence of different types of noises. Dropout and cell count imbalance noises are two major types and the most challenging in scRNA-seq data. Dropout is a technical noise in the form of missing value in gene expression [10], while cell number imbalance among cell types is coming from biology itself. We found gCAnno achieved the highest and rather stable kappa coefficients for both reference dropout and query dropout tests in four datasets (Fig. 4; Additional file 15: Figure S9; Additional file 16: Table S6; Additional file 17: Figure S10). Remarkably, gCAnno achieved average kappa coefficients of 0.88 (gCAnno-SVM) and 0.79 (gCAnno-Bayes) even when dropout rate was as high as 50%, while other methods achieve 0 (Scmap-cluster), 0.44 (Scmap-cell), 0.37 (Chetah), 0.25 (scPred) and 0.79 (SVM), respectively. Moreover, we found gCAnno, SVM and Scmap-cell achieved the highest and stable kappa coefficients (average values are about 0.99) for different cell count imbalance ratios (Additional file 15: Figure S9; Additional file 18: Table S7). All of these results show gCAnno is better than other methods for dropout and cell count imbalance noises and achieved the best performance on highly noisy data (e.g. 50% dropout rate and 1:0.1 imbalance rate), suggesting the effectiveness of the wCGBG in selecting accurate features in the presence of high noise.

### Cross platform annotation

Different single cell sequencing platforms have platform specific features or bias [19], limiting cross platform cell type annotation. We evaluated the platform compatibility of gCAnno on two liver datasets [4, 20] and two pancreas datasets [3, 21] from four platforms (10x, mCel-seq2, Drop-seq, and Smart-seq2) (Table 2). We used one platform dataset as the training data and the other as the testing data. For the performance comparison, gCAnno achieved consistently high kappa coefficient values for liver dataset tests (Fig. 5a and b) and for pancreas dataset tests (Fig. 5c and d) (Additional file 19: Table S8). These results show gCAnno is able to maintain high annotation accuracy for real heterogeneous and cross platform data in the presence of systematic platform specific bias.

**Table 2:** The list of scRNA-seq datasets in cross platform test

Dataset	#Cells	#Genes	# Cell types	Platform
Liver [4]	8103	20,007	7	10x
Pancreas [3]	8037	20,126	9	Drop-seq
Liver [20]	7130	33,941	7	mCel-seq2
Pancreas [21]	2068	25,526	9	Smart-seq2

# means the number of

**Fig. 5:**
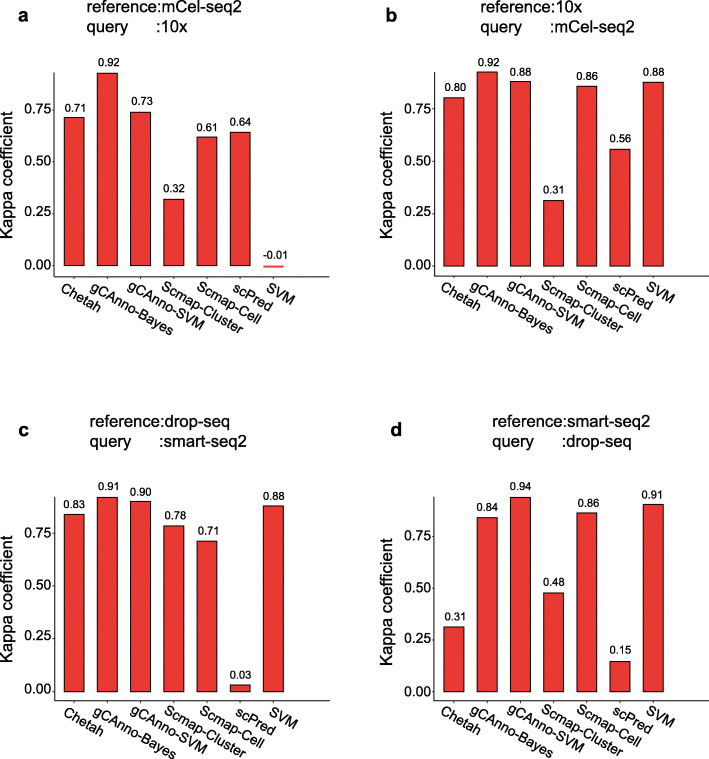
Platform compatibility evaluation. Performance comparisons of gCAnno with Scmap-Cluster, Scmap-Cell, scPred, Chetah and SVM on cross platform datasets. **a** liver datasets, where reference is mCel-seq2 and query is 10x; **b** liver datasets, where reference is 10x and query is mCel-seq2; **c** pancreas dataset, where reference is drop-seq and query is smart-seq2 **d** pancreas datasets, where reference is smart-seq2 and query is drop-seq. The reference is the training data and the query is the testing data

### Runtime evaluation

Finally, we evaluated the runtime of gCAnno based on datasets in above tests (Additional file 20: Table S9; Additional file 21: Figure S11). We found that the time takes in model building (including graph construction and embedding) step is positive correlated with the number of graph nodes (Pearson’s correlation is 0.94). Once the model has been built, the annotation step only takes less than 1 min (e.g. for mCel-seq2 platform liver dataset with 8103 cells only takes 48 s).

## Discussion

In this study, we present gCAnno, a novel graph-based cell type identification method for scRNA-seq data. The most significant feature of gCAnno is the construction of wCGBG, enabling gCAnno to capture the global characteristics of association between cell types and genes. This feature allows gCAnno to detect accurate feature genes for each cell type, leading to accurate annotation results and robustness for different noise types and rates. In addition, gCAnno is able to annotate not only human scRNA-seq, but also plant scRNA-seq (e.g. Arabidopsis data) and its stable and high performance across two platforms.

gCAnno contains SVM version (gCAnno-SVM) and naïve Bayes version (gCAnno-Bayes). The SVM version takes into account the effect of expression value while naïve Bayes version only considers the existence of cell type specific genes. From the evaluation result, the SVM version seems suitable for the dataset with deep annotation level and contains largely similar cell types between training and test sets. However, in cross platform datasets from different studies and different sequencing platforms, gene expression value might fluctuate significantly, rendering better performance of naïve Bayes version than SVM version.

Since gCAnno is a reference-based cell type annotation method, it lacks the ability to identify novel cell types. For novel type cells, gCAnno assigns the closest cell types with the most similar expression profiles to them, which might be reasonable in most of applications but probably require further improvement. Integrating the biomarker-based method for novel cell type annotation and reference-based method for accurate pre-defined cell type annotation, we think, will be one direction to explore.

## Conclusion

We have implemented a stable and high-performance automated cell type annotation tool, gCAnno, for scRNA-seq datasets. With an easy use Python running script as an example, we hope gCAnno will be useful for the scRNA-seq data analysis.

## Methods

Here we summarized the framework of gCAnno. gCAnno adopts graph structure for cell type specific gene set detection and accurate cell type annotation. Firstly, gCAnno builds cell type-gene bipartite graph based on gene expression abundances and intensities, in which gene expression abundance is the proportion of cells expressing the gene in a given cell type while intensity is the average expression in cells expressing the gene. Then, graph embedding is adopted to obtain the embedding vectors of gene nodes and cell type nodes. Next, gCAnno selects a set of genes for each cell type with similar profiles in the embedding space. Finally, based on the detected cell type specific genes, gCAnno trains naïve Bayes and SVM classifiers. The workflow of gCAnno is depicted in Fig. 1.

### Cell type-gene bipartite graph construction

Starting from the well-annotated reference scRNA-seq data, we constructed a weighted cell type-gene bipartite graph (wCGBG) containing both cell type nodes (CTN) and gene nodes (GN). Edges between CTN and GN indicate the correlation of a gene and a cell type while weight *W* measures significance of correlation. The weight is calculated by:

1 $$ {w}_{k,j}=\Big\{{\displaystyle \begin{array}{l}\frac{m_{k,j}}{n_k}\times mean\left(\overrightarrow{g_{k,j}}\right),\kern2em if\kern1em {n}_k\ne 0\\ {}0\kern7.8em ,\kern2em others\end{array}}\operatorname{} $$

where *n*_*k*_ is the cell count of cell type *k*, *m*_*j*, *k*_ is the number of cells expressed gene *j* in cell type *k*. $\overrightarrow{g_{j,k}}$ is the expression vector of gene *j* in cell type *k*. *W* is the product of the gene expression abundance and intensity. We use gene expression abundance and intensity to establish a relationship between cell types and genes in the form of proportion to reduce the impact of individual gene loss (dropout) or cell number imbalance.

### Graph embedding and cell type-gene specific relation detection

After wCGBG construction, we used node2vec to obtain the low dimensional vectors (the embedding vectors) of gene nodes and cell type nodes. The first step is construction of a neighborhood set *N*(*u*) of each node *u* (either gene or cell type node) by a probability walk [22]. Then, we optimized the following objective function *f*(*u*) by maximizing the log-probability of observing a neighborhood set.

2 $$ {\max}_f\sum \limits_{u\in V}\log P\left(N(u)|f(u)\right) $$

This optimization step enables the embedding vectors to capture the specificity and strength of interactions between cell node and gene node, e.g. if one gene is specific and highly expressed in one cell type, the corresponding two embedding vectors are similar. Then, we calculated Euclidean distance between the vector of genes and cell types. We selected top *n* (a user defined parameter, default *n* = 65, Additional file 1: Figure S1) closest genes for each cell type as the cell type specific gene set based on the overall performance on the five datasets we used [3–6, 18].

### Classifier construction

After obtaining the cell type specific gene set, we build naïve Bayes (gCAnno-Bayes) and SVM (gCAnno-SVM) classifiers for annotation. For gCAnno-SVM, we directly use the expression of cell type specific genes as features to train an SVM classifier. For gCAnno-Bayes, we build a binary matrix to presents cell type and its corresponding specific genes, e.g. the element *b*_*ij*_ = 1 indicates gene *j* is one of the specific genes in cell type *i*. We train a Bernoulli Naïve Bayes to get genes’ conditional probability in each cell type and the prior probability of cell types. The query dataset is binarized and the annotation is based on maximum posterior probability of single cell’s cell type specific genes expression.

### Performance measurement and dataset

#### Performance assessment and comparison

Cell type annotation is a typical multi-classification problem. We applied kappa coefficient as the performance measurement of classification, defined as Eq. (3).

3 $$ \kappa =\frac{p_o-{p}_e}{1-{p}_e},\kern0.5em {p}_o=\frac{N_{corr}}{N_t},\kern0.5em {p}_e=\frac{\sum \limits_{i=1}^K{a}_i\times {b}_i}{N_t\times {N}_t} $$

where *N*_corr_ is the ratio of total number of cells with corrected cell type annotation, *N*_t_ is the total number of cells in the dataset, *K* is the number of truly cell types, *a*_*i*_ is the number of corrected annotated cells in the *i*-th cell type, and *b*_*i*_ is the number of cells in the *i*-th cell type, *p*_*o*_ is the accuracy, *a*_*i*_ × *b*_*i*_ is the product of the actual and predicted quantity, *p*_*e*_ punishes bias for unbalance evaluation.

To evaluate the performance of gCAnno, we performed both cross-validation test and independent heterogeneous test (cross-platform test). First, we adopted the five-fold cross-validation strategy following recent single cell analysis comparison published earlier [15, 17] on four published datasets and simulated noise datasets to evaluate the overall and robustness performance (Additional file 2: File S1). Then, we performed independent test on datasets from different sequencing platforms (the cross-platform testing) to evaluate the generalization capability of gCAnno.

#### Tools in comparison

The calculation results of Scmap, Chetah and scPred were obtained from the corresponding publications [13–15]. For SVM, we followed the previous report [17] which is using drop-based method [23] for feature selection.

#### Datasets used in basic overall performance test

To illustrate the stable performance of gCAnno across various species and tissue types, we compared gCAnno with other methods using four published datasets, including liver, pancreas, *Arabidopsis thaliana* root (AT root), hepatocellular carcinoma and intrahepatic cholangiocarcinoma (HCC and ICCA) datasets (Table 1; Additional file 2: File S1; Additional file 3: Figure S2; Additional file 4: Table S1). The true labels of the cells in each dataset are obtained from the corresponding publications.

#### Large dataset with deep annotation level

To demonstrate the performance of gCAnno in large dataset (cell number more than 50,000) with deep annotation level (more than 20 cell types). We compared gCAnno with other methods in 20 mouse organs dataset with 54,246 cells, 29 cell types and 23,433 genes. The true labels of the cells in each dataset are also obtained from the original publications [18] (Additional file 2: File S1; Additional file 5: Figure S3; Additional file 6: Table S2).

#### Simulated dropout and imbalance datasets

To evaluate the robustness of gCAnno in the presence of dropout noise, we simulated different dropout rates in four above datasets (Table 1), by modifying the expression level of a random gene subset (10, 20, 30, 40 and 50% of all genes) to zero (Additional file 2: File S1). Similarly, we used five-fold cross validation to evaluate its performance. In each validation, we simulated the dropout noise in either training group (reference dropout) or test group (query dropout), and calculated the kappa coefficient for each method.

To simulate the cell number imbalance noise, we randomly sampled different proportions (0.1:1, 0.3:1, 0.5:1, 0.7:1, 0.9:1, 1:0.9, 1:0.7, 1:0.5, 1:0.3 and 1:0.1) of cell count in two cell types (Hepatocyte and GamaDetaT) in liver dataset as the reference data for classifier constructing. To get more accuracy testing, this simulation was repeated five times (Additional file 2: File S1).

#### Cross platform datasets

To compare cross platform performance (various studies using different sequencing platforms), we searched and identified four datasets suitable for this purpose, including two liver datasets from 10x and mCel-seq2 platforms and two pancreas datasets from drop-seq and smart-seq2 platforms (Table 2). We noticed that the cell type annotation labels of the same tissue from different platforms are not identical. Thus, we unified the labels by removing cell types absent in either of the datasets (Additional file 7: Figure S4; Additional file 8: Table S3; Additional file 2: File S1).

## Supplementary Information

**Additional file 1:** **Figure S1.** The test of gCAnno parameter top closest genes in five evaluation datasets. The parameter is stable in 25 to 85. When top gene select less than 5 (in all datasets) and more than 125 (in Arabidopsis and liver datasets), the performance are not well. In our evaluation, the default top closest genes in each cell type is 65 and user can adjustment by themselves.

**Additional file 2:** **File S1.** Supplementary Materials, including data preparation, cell type information of each datasets, and supplementary methods.

**Additional file 3:** **Figure S2.** The tSNE plot of (a) liver, (b) pancreas, (c) HCC & ICCA and (d) AT root datasets.

**Additional file 4:** **Table S1.** The tSNE result, cell barcodes and cell type labels of (a) liver, (b) pancreas, (c) HCC & ICCA and (d) AT root datasets.

**Additional file 5:** **Figure S3.** The tSNE plot of a large dataset with deep annotation level (20 mouse organs).

**Additional file 6:** **Table S2.** The large dataset kappa coefficient result (Fig. 2e) and tSNE result.

**Additional file 7:** **Figure S4.** The tSNE plot of (a) mCel-seq2 liver, (b) 10x liver, (c) Drop-seq pancreas and (d) Smart-seq2 pancreas.

**Additional file 8:** **Table S3.** The tSNE result, cell barcodes and cell type labels of (a) mCel-seq2 liver, (b) 10x liver, (c) Drop-seq pancreas and (d) Smart-seq2 pancreas.

**Additional file 9:** **Figure S5.** The tSNE of embedding vectors of cell type nodes and gene nodes in four datasets. The selecting gene nodes are in red color and non-selecting gene nodes are in grey. The cell type nodes are blue triangles.

**Additional file 10:** **Figure S6.** An example of top 2 specific genes in each cell type of liver dataset. In tSNE plot, each gene specific expressed in corresponding cell type in red color. The shade of color means the expression value.

**Additional file 11:** **Figure S7.** The overlap of reported marker genes from the corresponding publications in four datasets with selected genes. The circle is selected genes and the square is not selected genes. The marker genes have different color and non-marker genes are gray.

**Additional file 12:** **Table S4.** The statistic of select state of reported marker genes from the corresponding publications in four datasets with selected genes.

**Additional file 13:** **Table S5.** The statistic of kappa coefficient in overall performance test (Fig. 2a-d).

**Additional file 14:** **Figure S8.** The heatmap of each cell type specific genes expression in large dataset (top closest gene number 65). It shows an obvious pattern in diagonal.

**Additional file 15:** **Figure S9.** Comparisons of gCAnno with Scmap-Cluster, Scmap-Cell, scPred, Chetah and SVM on (a) HCC and ICCA reference dropout dataset, (b) HCC and ICCA query dropout dataset, (c) AT root reference dropout dataset, (d) AT root query dropout dataset and (e) imbalance dataset.

**Additional file 16:** **Table S6.** The statistic of kappa coefficient in dropout test.

**Additional file 17:** **Figure S10.** An example of the existence of selected cell type specific genes in liver ref. dropout test dataset. The red color in more than one type means these types shared this gene. With the increasing of dropout rate, the degree of shared specific genes increased a little, but the specific pattern is still strong even in dropout rate 50%.

**Additional file 18:** **Table S7.** The statistic of kappa coefficient in imbalance test.

**Additional file 19:** **Table S8.** The statistic of kappa coefficient in cross platform test.

**Additional file 20:** **Table S9.** Runtime statistic of gCAnno.

**Additional file 21:** **Figure S11.** The plot of building model time and graph scale. The building model time is correlated with graph node number (correlation coefficient is 0.94).

## Data Availability

Datasets used for the analyses in this study are summarized in Additional file 2: File S1. The source code of gCAnno is publicly available at https://github.com/xjtu-omics/gCAnno.

## Abbreviations

kappa: Kappa coefficient
DEGs: Differentially expressed genes
UMI: Unique Molecular Identifier
scRNA-seq: Single-cell RNA-seq
PCs: Principle components
HCA: Human cell atlas
wCGBG: Weighted cell type-gene bipartite graph
10x: 10x Genomics platform
SVM: Support Vector Machine

## References

[1] Kolodziejczyk AA, Kim JK, Svensson V, Marioni JC, Teichmann SA (2015). The technology and biology of single-cell RNA sequencing. Mol Cell.

[2] Tang F, Barbacioru C, Wang Y, Nordman E, Lee C, Xu N, Wang X, Bodeau J, Tuch BB, Siddiqui A (2009). mRNA-Seq whole-transcriptome analysis of a single cell. Nat Methods.

[3] Baron M, Veres A, Wolock SL, Faust AL, Gaujoux R, Vetere A, Ryu JH, Wagner BK, Shen-Orr SS, Klein AM (2016). A single-cell transcriptomic map of the human and mouse pancreas reveals inter- and intra-cell population structure. Cell Syst.

[4] MacParland SA, Liu JC, Ma XZ, Innes BT, Bartczak AM, Gage BK, Manuel J, Khuu N, Echeverri J, Linares I (2018). Single cell RNA sequencing of human liver reveals distinct intrahepatic macrophage populations. Nat Commun.

[5] Ma L, Hernandez MO, Zhao Y, Mehta M, Tran B, Kelly M, Rae Z, Hernandez JM, Davis JL, Martin SP (2019). Tumor cell biodiversity drives microenvironmental reprogramming in liver cancer. Cancer Cell.

[6] Zhang TQ, Xu ZG, Shang GD, Wang JW (2019). A single-cell RNA sequencing profiles the developmental landscape of Arabidopsis root. Mol Plant.

[7] Butler A, Hoffman P, Smibert P, Papalexi E, Satija R (2018). Integrating single-cell transcriptomic data across different conditions, technologies, and species. Nat Biotechnol.

[8] Wolf FA, Angerer P, Theis FJ (2018). SCANPY: large-scale single-cell gene expression data analysis. Genome Biol.

[9] Guo M, Wang H, Potter SS, Whitsett JA, Xu Y (2015). SINCERA: a pipeline for single-cell RNA-Seq profiling analysis. PLoS Comput Biol.

[10] Kiselev VY, Andrews TS, Hemberg M (2019). Challenges in unsupervised clustering of single-cell RNA-seq data. Nat Rev Genet.

[11] Pliner HA, Shendure J, Trapnell C (2019). Supervised classification enables rapid annotation of cell atlases. Nat Methods.

[12] Zhang AW, O'Flanagan C, Chavez EA, Lim JLP, Ceglia N, McPherson A, Wiens M, Walters P, Chan T, Hewitson B (2019). Probabilistic cell-type assignment of single-cell RNA-seq for tumor microenvironment profiling. Nat Methods.

[13] Kiselev VY, Yiu A, Hemberg M (2018). scmap: projection of single-cell RNA-seq data across data sets. Nat Methods.

[14] de Kanter JK, Lijnzaad P, Candelli T, Margaritis T, Holstege FCP (2019). CHETAH: a selective, hierarchical cell type identification method for single-cell RNA sequencing. Nucleic Acids Res.

[15] Alquicira-Hernandez J, Sathe A, Ji HP, Nguyen Q, Powell JE (2019). scPred: accurate supervised method for cell-type classification from single-cell RNA-seq data. Genome Biol.

[16] Regev A, Teichmann SA, Lander ES, Amit I, Benoist C, Birney E, Bodenmiller B, Campbell P, Carninci P, Clatworthy M (2017). The human cell atlas. Elife.

[17] Abdelaal T, Michielsen L, Cats D, Hoogduin D, Mei H, Reinders MJT, Mahfouz A (2019). A comparison of automatic cell identification methods for single-cell RNA sequencing data. Genome Biol.

[18] Tabula Muris C, Overall c, Logistical c, Organ c, processing, Library p, sequencing, Computational data a, Cell type a, Writing g (2018). Single-cell transcriptomics of 20 mouse organs creates a Tabula Muris. Nature.

[19] Ziegenhain C, Vieth B, Parekh S, Reinius B, Guillaumet-Adkins A, Smets M, Leonhardt H, Heyn H, Hellmann I, Enard W (2017). Comparative analysis of single-cell RNA sequencing methods. Mol Cell.

[20] Aizarani N, Saviano A, Sagar, Mailly L, Durand S, Herman JS, Pessaux P, Baumert TF, Grun D (2019). A human liver cell atlas reveals heterogeneity and epithelial progenitors. Nature.

[21] Segerstolpe A, Palasantza A, Eliasson P, Andersson EM, Andreasson AC, Sun X, Picelli S, Sabirsh A, Clausen M, Bjursell MK (2016). Single-cell transcriptome profiling of human pancreatic islets in health and type 2 diabetes. Cell Metab.

[22] Grover A, Leskovec J (2016). node2vec: scalable feature learning for networks. KDD.

[23] Andrews TS, Hemberg M (2019). M3Drop: dropout-based feature selection for scRNASeq. Bioinformatics.

