# Supplementary figures and images for: gCAnno: a graph-based single cell type annotation method
Source: BMC Genomics. 2020 Nov 23;21:823. doi: 10.1186/s12864-020-07223-4 (PMC7686723; doi:10.1186/s12864-020-07223-4)

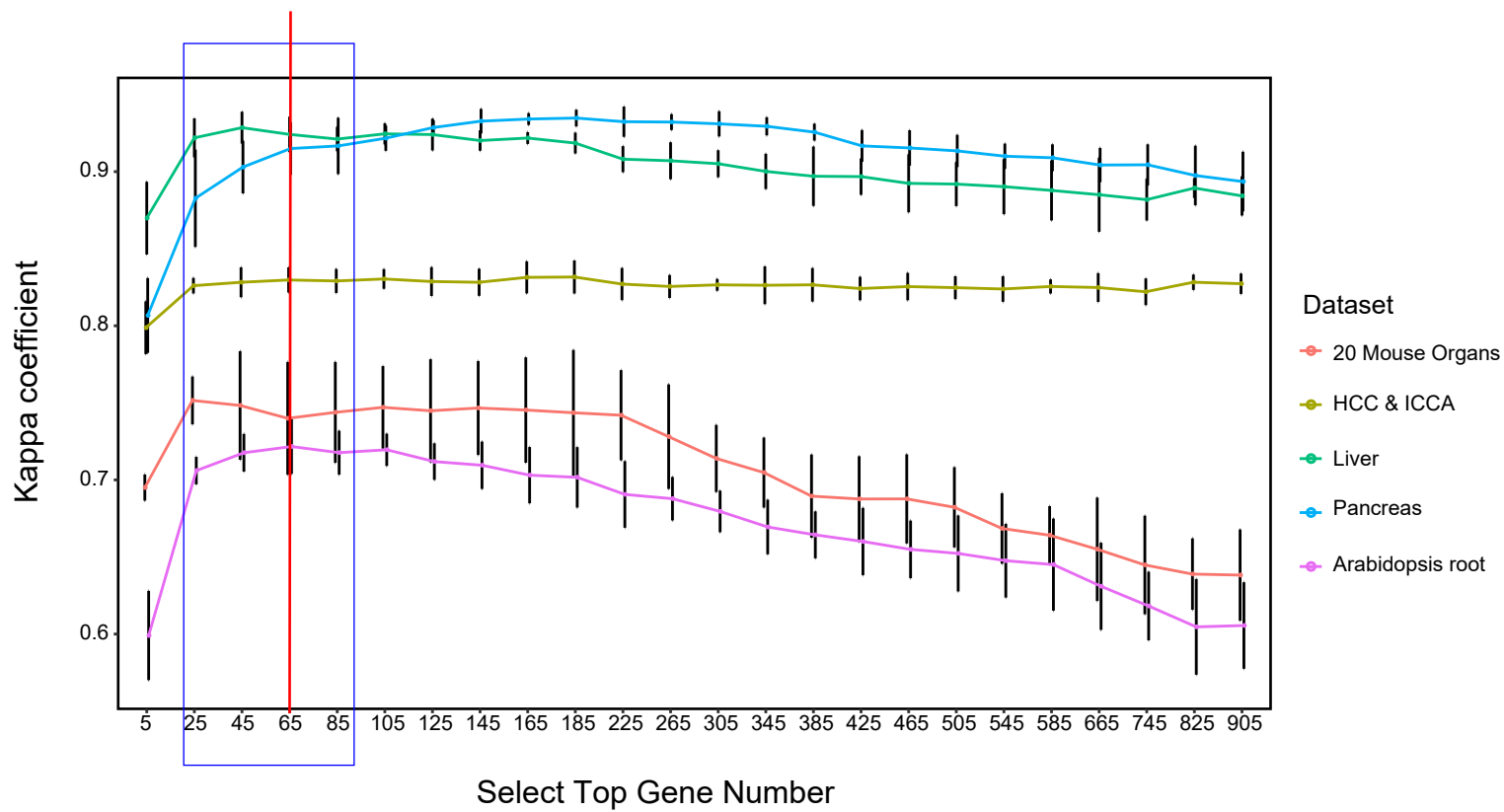

Supplement: Supplementary file 1 — Additional file 1: Figure S1. The test of gCAnno parameter top closest genes in five evaluation datasets. The parameter is stable in 25 to 85. When top gene select less than 5 (in all datasets) and more than 125 (in Arabidopsis and liver datasets), the performance are not well. In our evaluation, the default top closest genes in each cell type is 65 and user can adjustment by themselves. [file 12864_2020_7223_MOESM1_ESM.pdf]

A

Liver

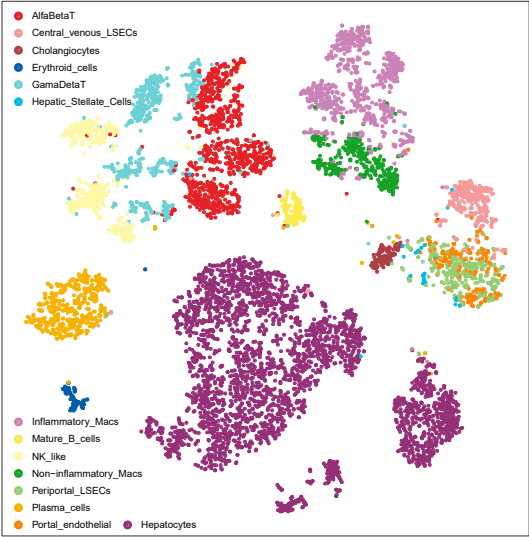

B

Pancreas

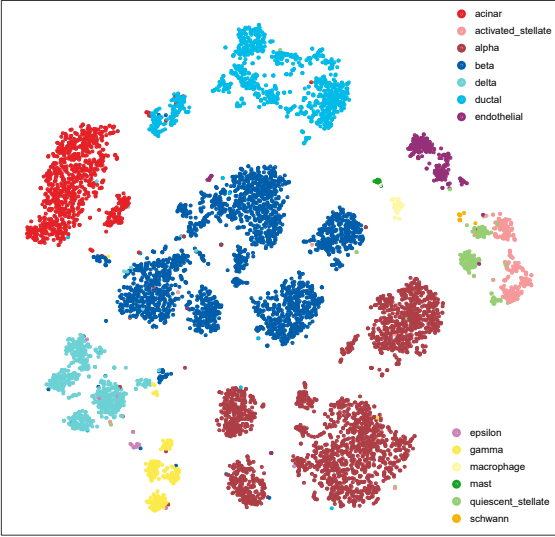

C

HCC & ICCA

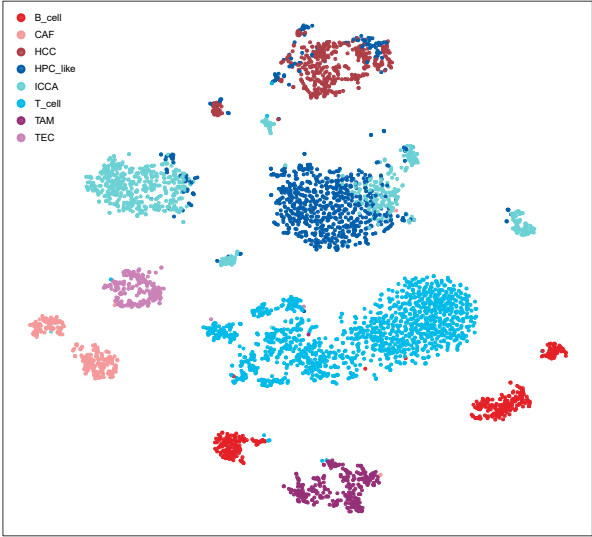

D

Arabidopsis thaliana root

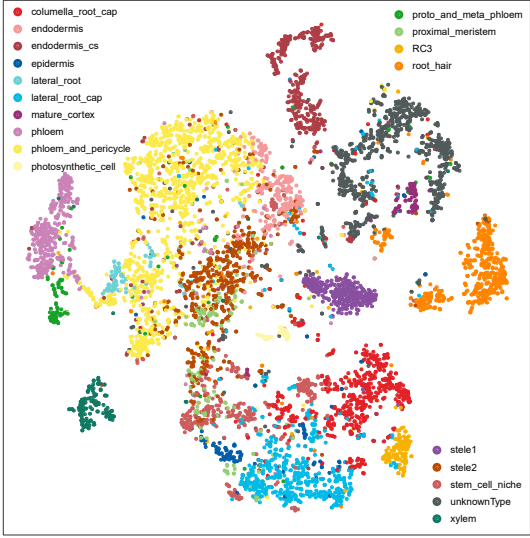

Supplement: Supplementary file 3 — Additional file 3: Figure S2. The tSNE plot of (a) liver, (b) pancreas, (c) HCC & ICCA and (d) AT root datasets. [file 12864_2020_7223_MOESM3_ESM.pdf]

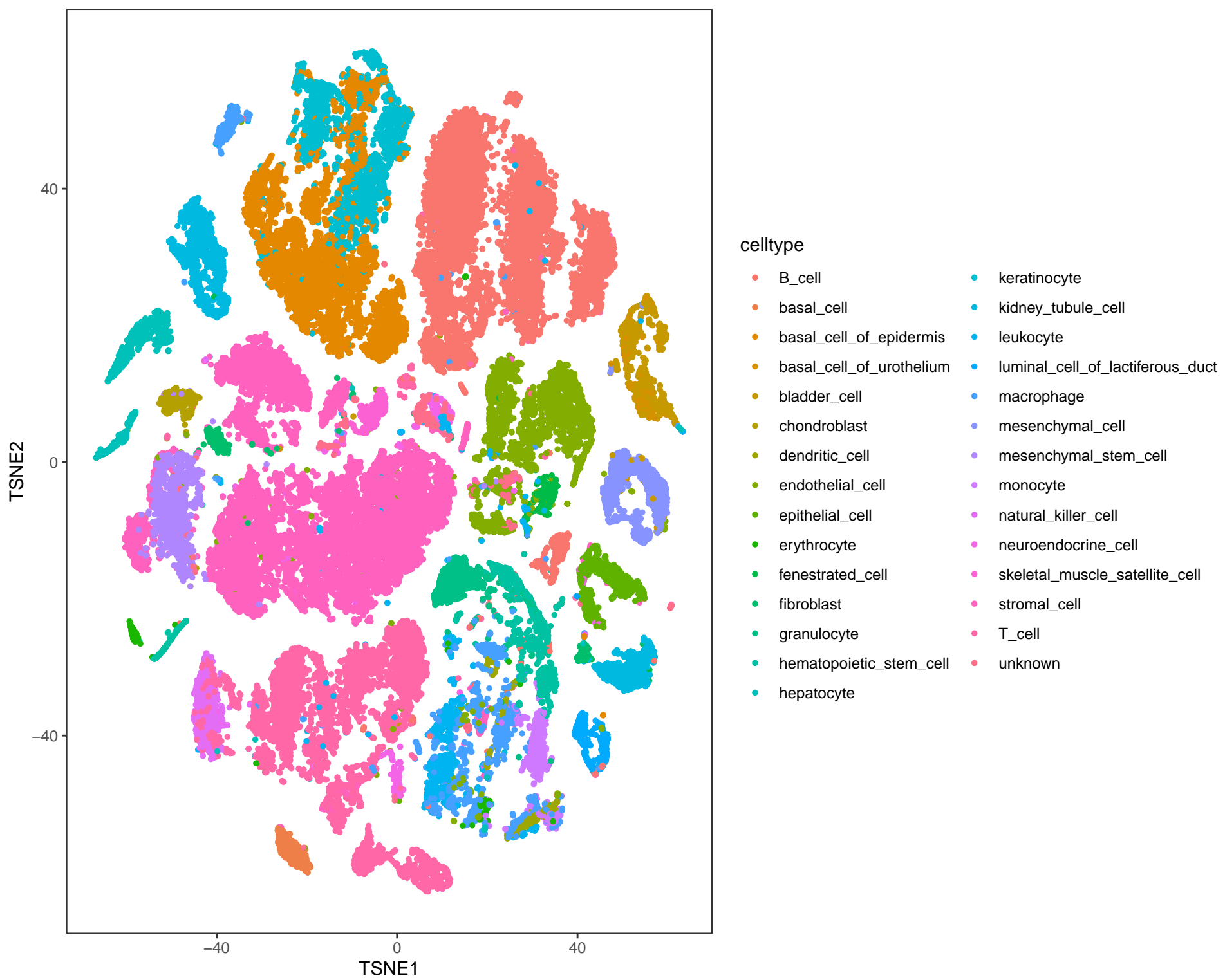

Supplement: Supplementary file 5 — Additional file 5: Figure S3. The tSNE plot of a large dataset with deep annotation level (20 mouse organs). [file 12864_2020_7223_MOESM5_ESM.pdf]

## A Liver mCel-seq2

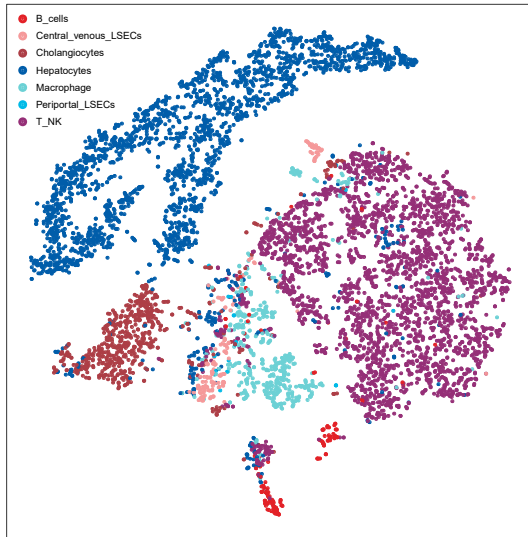

## B Liver 10x

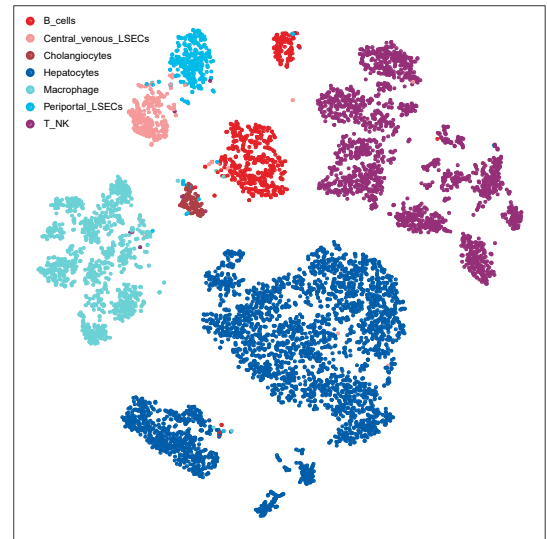

## C Pancreas Drop-seq

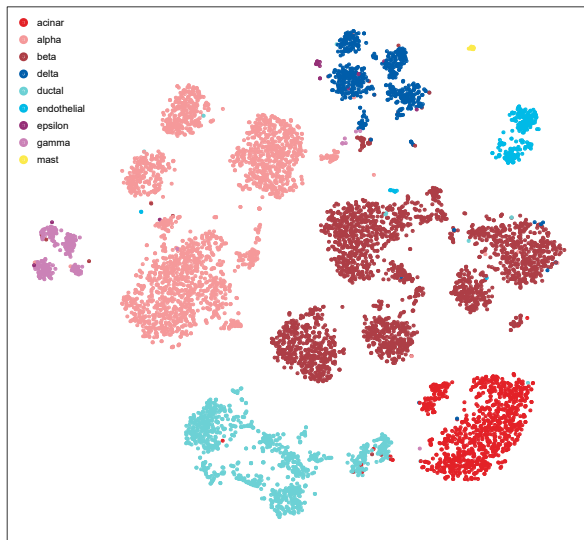

## D Pancreas Smart-seq2

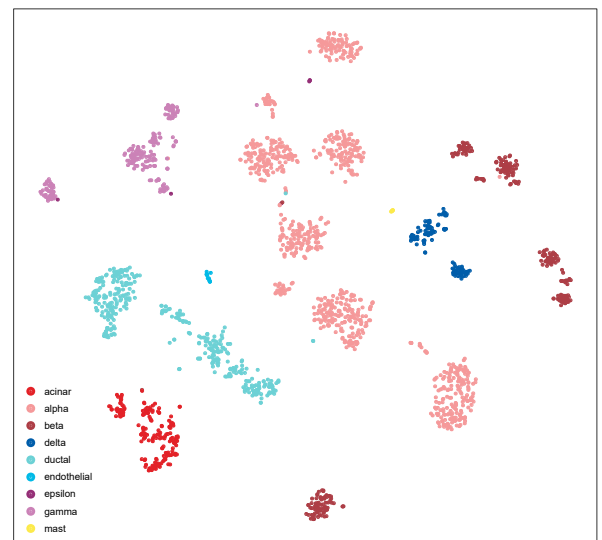

Supplement: Supplementary file 7 — Additional file 7: Figure S4. The tSNE plot of (a) mCel-seq2 liver, (b) 10x liver, (c) Drop-seq pancreas and (d) Smart-seq2 pancreas. [file 12864_2020_7223_MOESM7_ESM.pdf]

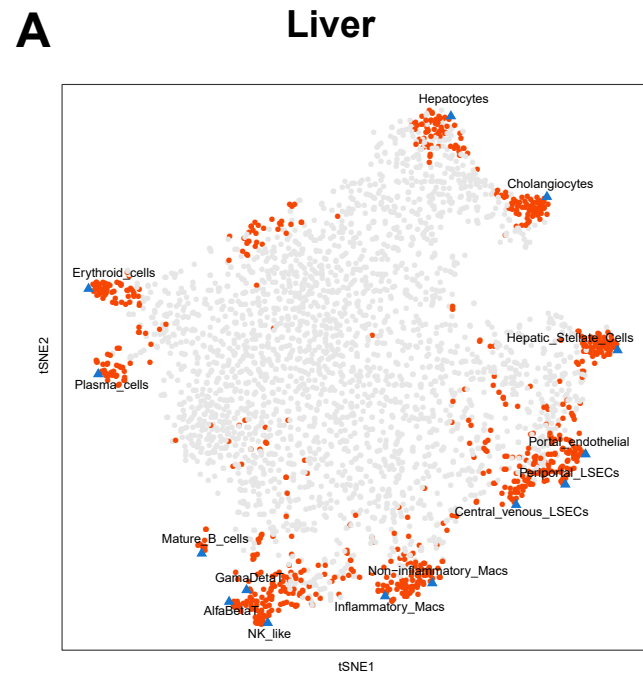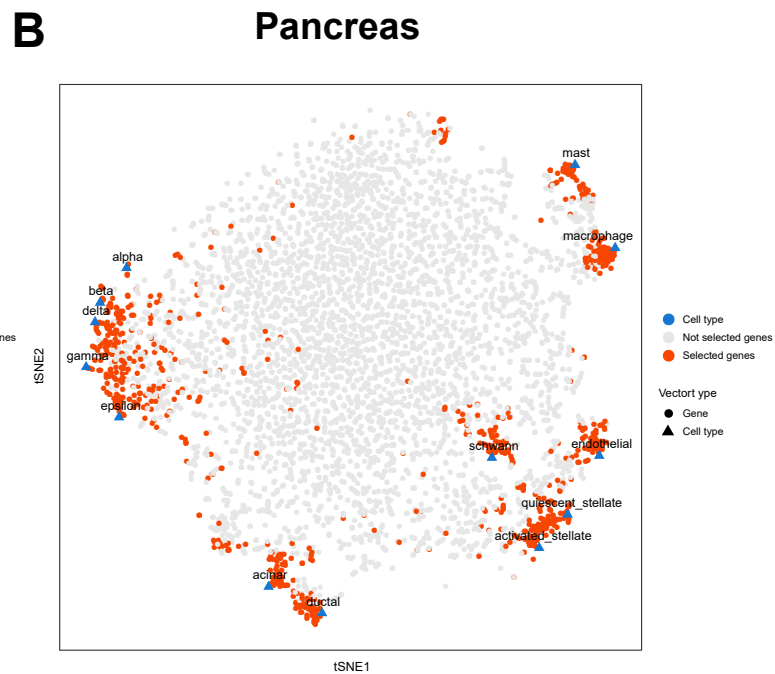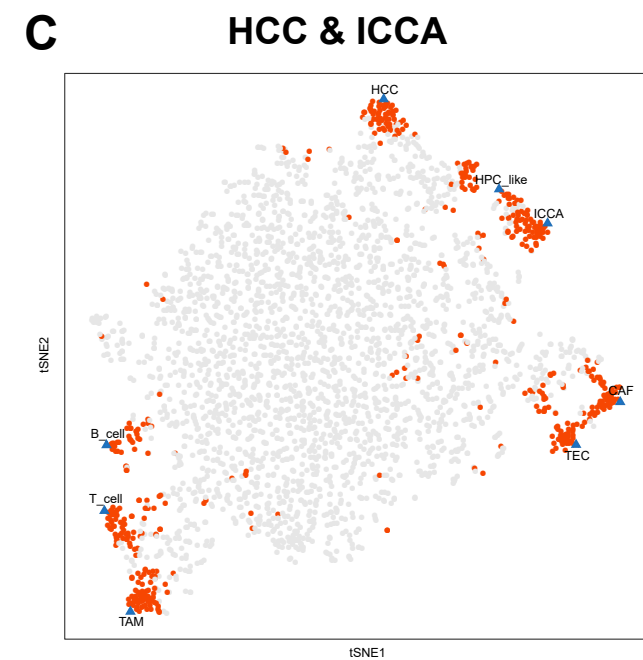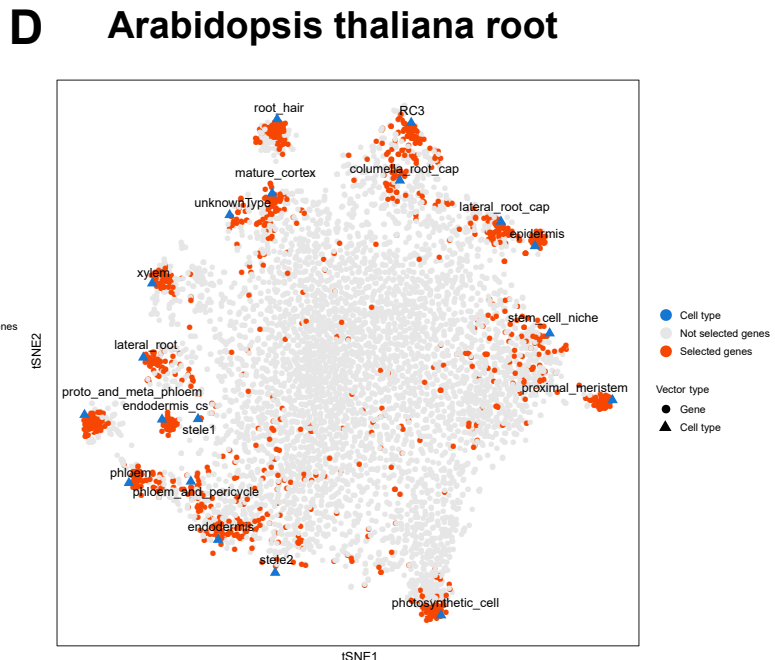

Supplement: Supplementary file 9 — Additional file 9: Figure S5. The tSNE of embedding vectors of cell type nodes and gene nodes in four datasets. The selecting gene nodes are in red color and non-selecting gene nodes are in grey. The cell type nodes are blue triangles. [file 12864_2020_7223_MOESM9_ESM.pdf]

**A****Liver**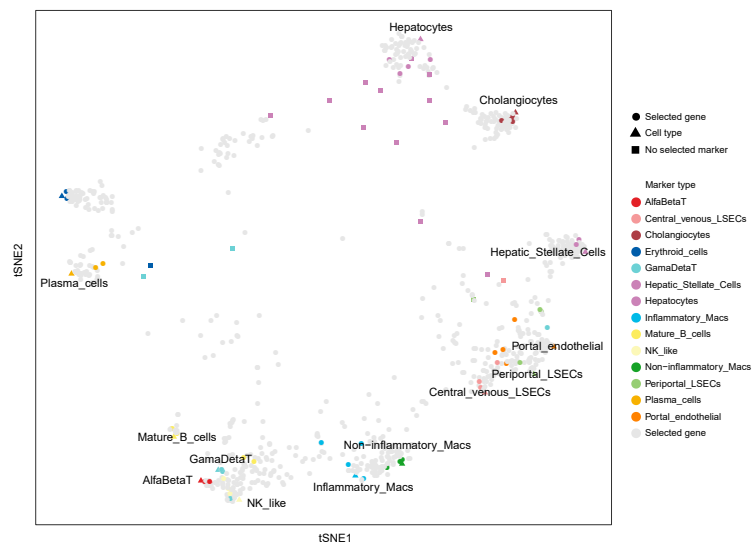**B****Pancreas**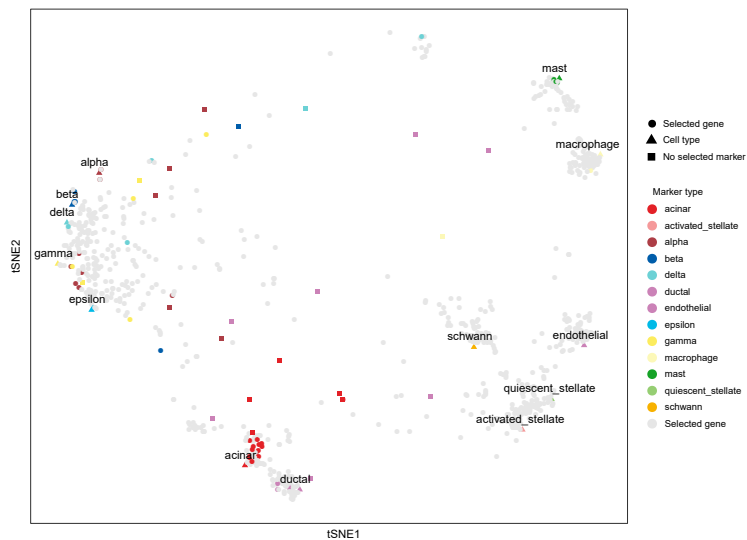**C****HCC & ICCA**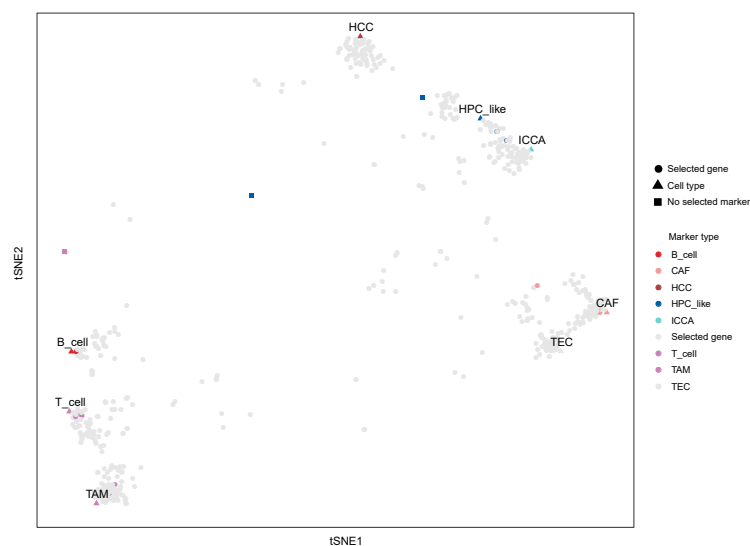**D****Arabidopsis thaliana root**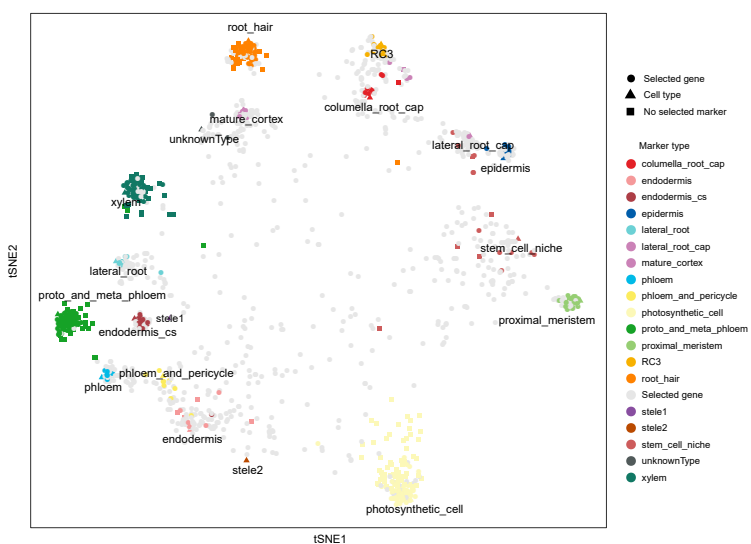

Supplement: Supplementary file 11 — Additional file 11: Figure S7. The overlap of reported marker genes from the corresponding publications in four datasets with selected genes. The circle is selected genes and the square is not selected genes. The marker genes have different color and non-marker genes are gray. [file 12864_2020_7223_MOESM11_ESM.pdf]

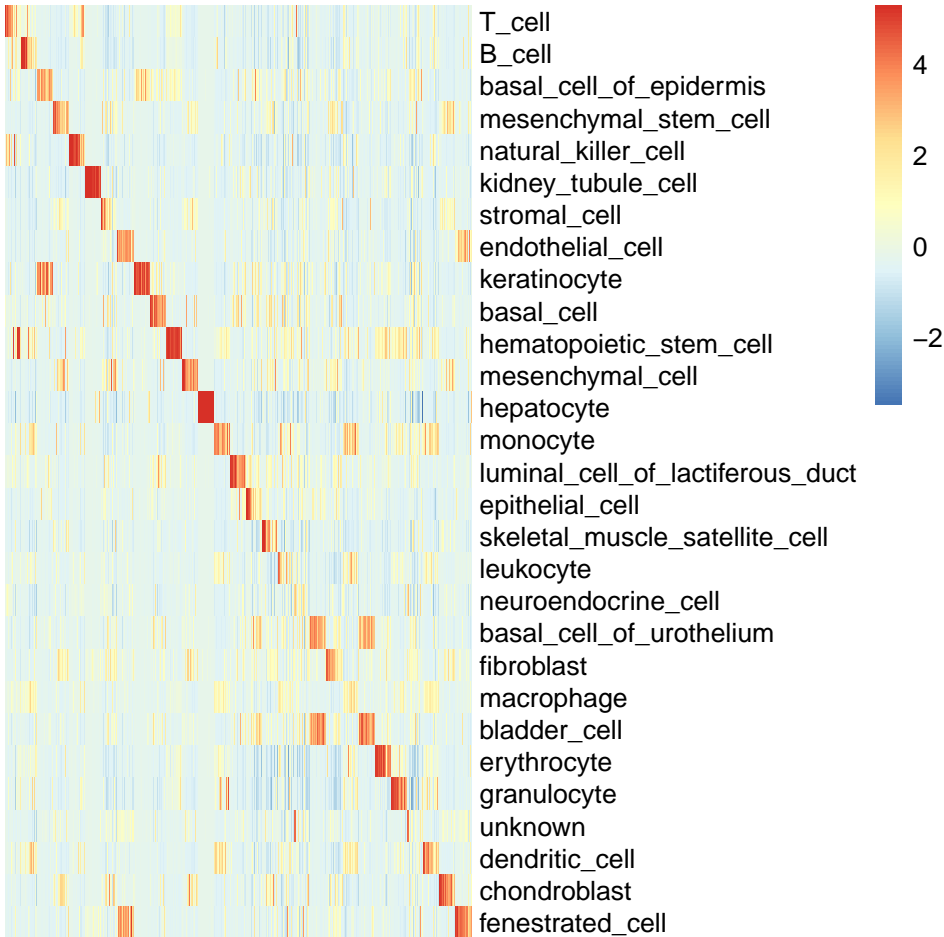

Supplement: Supplementary file 14 — Additional file 14: Figure S8. The heatmap of each cell type specific genes expression in large dataset (top closest gene number 65). It shows an obvious pattern in diagonal. [file 12864_2020_7223_MOESM14_ESM.pdf]

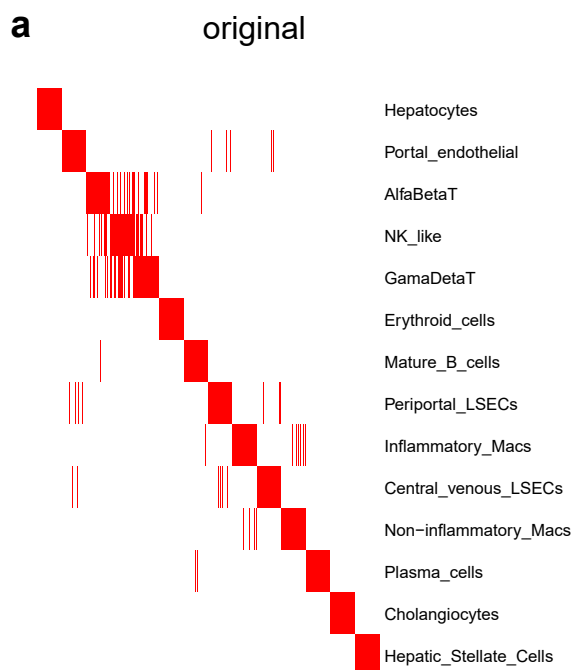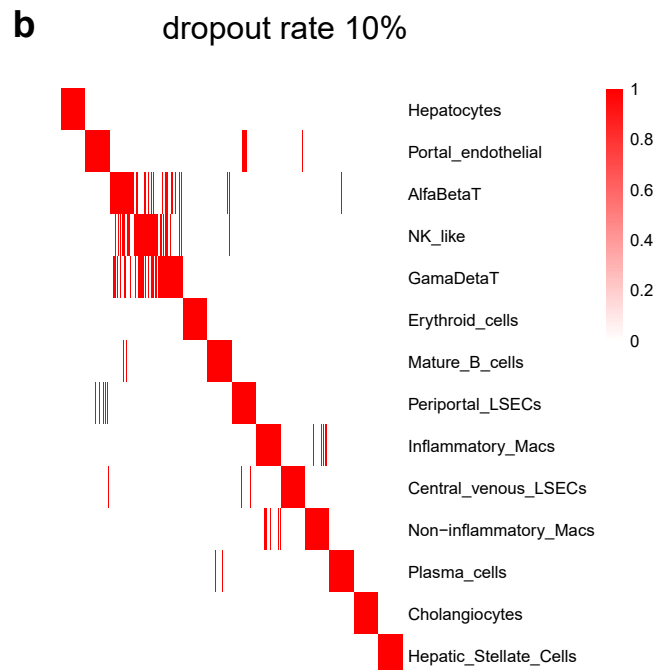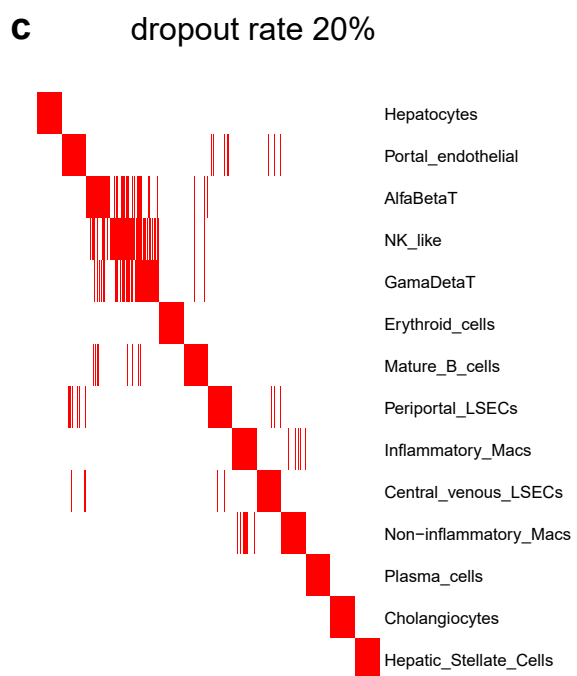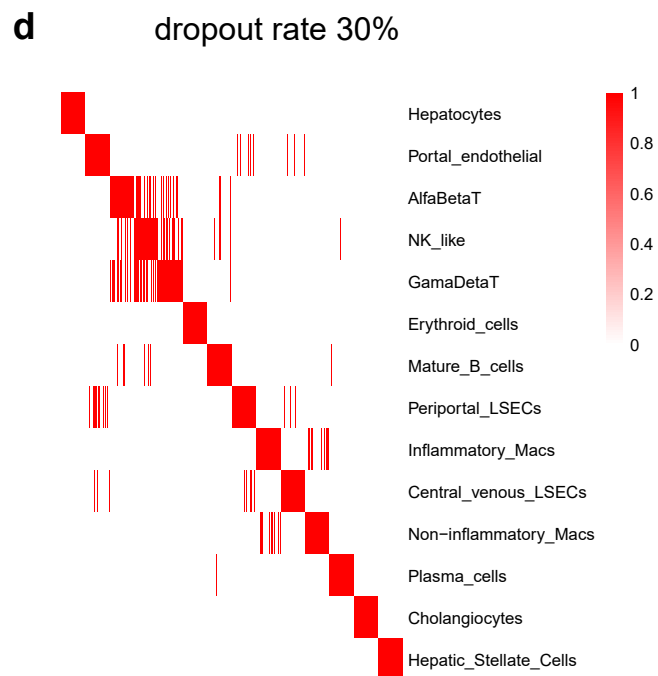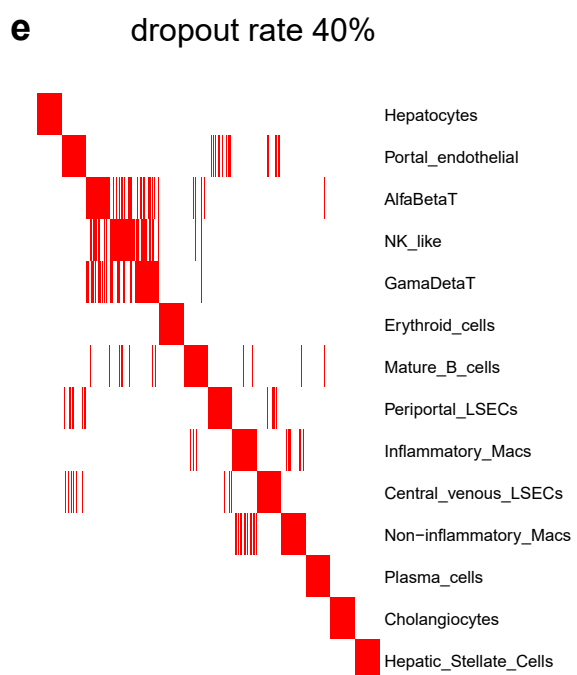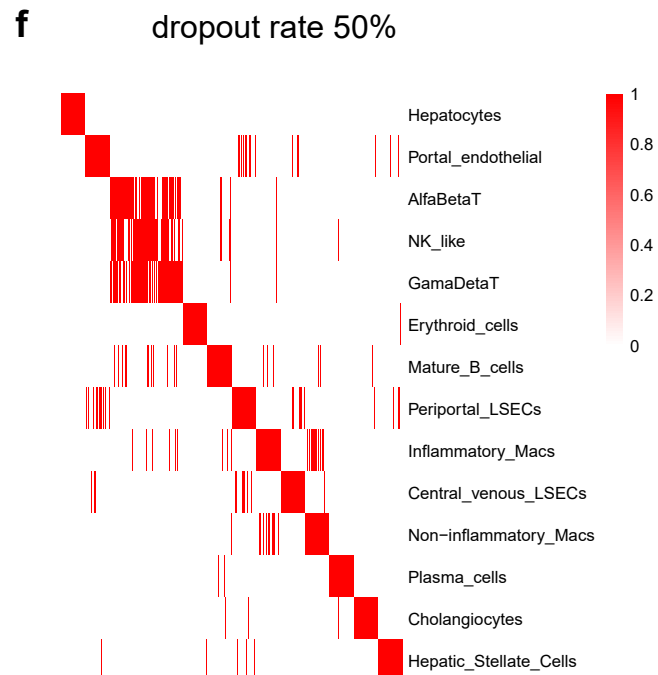

Supplement: Supplementary file 17 — Additional file 17: Figure S10. An example of the existence of selected cell type specific genes in liver ref. dropout test dataset. The red color in more than one type means these types shared this gene. With the increasing of dropout rate, the degree of shared specific genes increased a little, but the specific pattern is still strong even in dropout rate 50%. [file 12864_2020_7223_MOESM17_ESM.pdf]

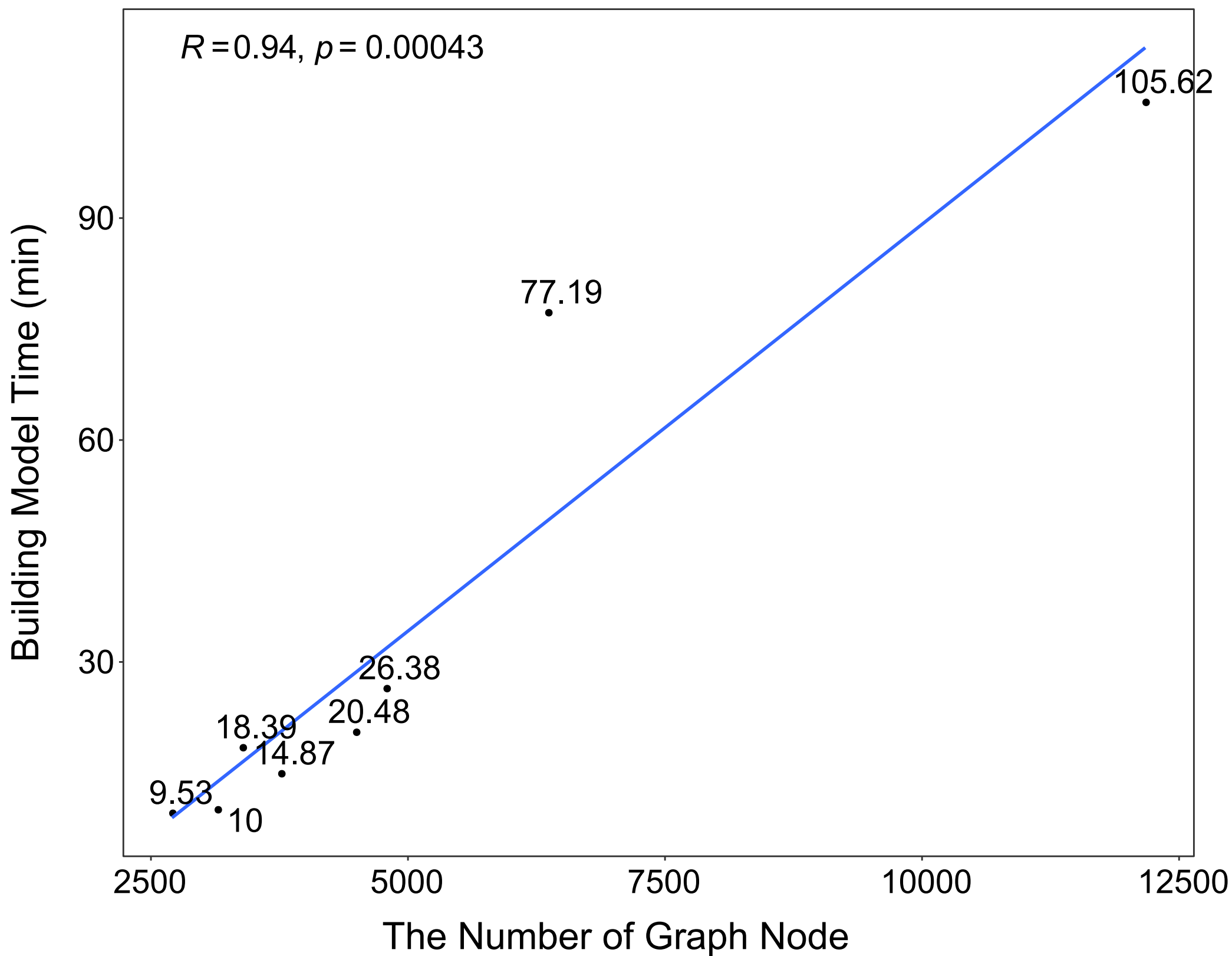

Supplement: Supplementary file 21 — Additional file 21: Figure S11. The plot of building model time and graph scale. The building model time is correlated with graph node number (correlation coefficient is 0.94). [file 12864_2020_7223_MOESM21_ESM.pdf]
