# Supplementary material for: gCAnno: a graph-based single cell type annotation method
Source: BMC Genomics. 2020 Nov 23;21:823. doi: 10.1186/s12864-020-07223-4 (PMC7686723; doi:10.1186/s12864-020-07223-4)
Supplement: Supplementary file 2 — Additional file 2: File S1. Supplementary Materials, including data preparation, cell type information of each datasets, and supplementary methods. [file 12864_2020_7223_MOESM2_ESM.docx]

**Supplementary Materials for “gCAnno: a graph based cell type annotation method for single-cell RNA sequencing”**

**Supplementary Notes**

1. **Datasets preparation**

All final selected cell barcodes can be found in Additional file 4, Additional file 6 and Additional file 8. The detail of data set preparation is described below.

- 1. **Datasets used in overall performance test**

*Liver dataset [1]*

| Expression profile | The resource came from the original research which was normalized by cpm. |
| --- | --- |
| Cell type information | We got this information in the original research. In addition, we merged “Hep1”, “Hep2”, “Hep3”, “Hep4”, “Hep5” and “Hep6” into “Hepatocytes”, “γδ T cells 1” and “γδ T cells 1” into “GamaDetaT”. |
| Final types | “AlfaBetaT”, “Central_venous_LSECs”, “Cholangiocytes”, “Erythroid_cells”, “GamaDetaT”, “Hepatic_Stellate_Cells”, “Hepatocytes”, “Inflammatory_Macs”, “Mature_B_cells”, “NK_like”, “Non-inflammatory_Macs”, “Periportal_LSECs”, “Plasma_cells”, “Portal_endothelial” |

*Pancreas dataset [2]*

| Expression profile | The resource came from the original research which is count matrix. We used cpm to normalize the count matrix. |
| --- | --- |
| Cell type information | We got this information in the original research. |
| Final types | “acinar”, “activated_stellate”, “alpha”, “beta”, “delta”, “ductal”, “endothelial”, “epsilon”, “gamma”, “macrophage”, “mast”, “quiescent_stellate”, “schwann” |

*HCC, ICCA dataset [3]*

| Expression profile | We got the CellRanger (version 2.0.2) result count matrix from the original research. We filtered cells whose gene number lower than 100, umi count lower than 201 and the percentage of mitochondrial genes over than 20%. And then, we used cpm to normalize the count matrix. |
| --- | --- |
| Cell type information | We got this information in the original research. In addition, we divided “Malignant cell” into two categories “ICCA” and “HCC” based on the type of patient information which is offered by the original research. And we also removed the “unclassified” type. |
| Final types | “B_cell”, “CAF”, “HCC”, “HPC_like”, “ICCA”, “TAM”, “TEC”, “T_cell” |

*AT root dataset [4]*

| Expression profile | We got the fastq file from the original research. And then, we used the CellRanger (version 3.0.2) to get the count matrix and filtered cells whose gene number lower than 200 and umi count lower than 500. And then, we used cpm to normalize the count matrix. |
| --- | --- |
| Cell type information | We got the cluster information from the website which offered by the original research and cell type information from the article. |
| Final types | “RC3”, “columella_root_cap”, “endodermis”, “endodermis_cs”, “epidermis”, “lateral_root”, “lateral_root_cap”, “mature_cortex”, “phloem”, “phloem_and_pericycle”, “photosynthetic_cell”, “proto_and_meta_phloem”, “proximal_meristem”, “root_hair”, “stele1”, “stele2”, “stem_cell_niche”, “unknownType”, “xylem” |

*20 mouse organs [5]*

| Expression profile | The resource came from the original research which was normalized by cpm. |
| --- | --- |
| Cell type information | We got this information in the original research. In addition, we deleted cell types which cell number are less than 100. |
| Final types | “basal_cell”, “basal_cell_of_epidermis”, “basal_cell_of_urothelium”, “B_cell”, “bladder_cell”, “chondroblast”, “dendritic_cell”, “endothelial_cell”, “epithelial_cell”, “erythrocyte”, “fenestrated_cell”, “fibroblast”, “granulocyte”, “hematopoietic_stem_cell”, “hepatocyte”, “keratinocyte”, “kidney_tubule_cell”, “leukocyte”, “luminal_cell_of_lactiferous_duct”, “macrophage”, “mesenchymal_cell”, “mesenchymal_stem_cell”, “monocyte”, “natural_killer_cell”, “neuroendocrine_cell”, “skeletal_muscle_satellite_cell”, “stromal_cell”, “T_cell”, “unknown” |

- 1. **Datasets splitting for cross validation**

We adopted five-fold cross validation in performance test, following procedures used in published works [6]and [7]. For each dataset, we first randomly shuffled the dataset and split it into five nonoverlapped parts. In each validation, we considered one of the five parts as test data group and other four prats as training group. For each method, we trained the model on the training group data and calculate the kappa coefficient on the test group data. We repeated the validation five times to ensure every part will be the test group data to avoid bias.

- 1. **Simulated dropout and imbalance datasets.**
     1. Simulated dropout dataset

We simulated the dropout noise on four small dataset we used (Table 1), and we adopted five-fold cross validation to get the performance on data with dropout noise. Give a dataset, we simulated the dropout noise by setting the expression value of a randomly selected a percentage (10%, 20%, 30%, 40% or 50%) of genes in each cell to zero. For each dataset, in each validation, we first do the reference dropout test. We simulated dropout noise in training group data. We trained the model on the dropout noised training data and calculated kappa coefficients on the test data. Secondly, we do the query dropout test. We simulated dropout noise in test group data. We trained the model on training data (without dropout noise), and then calculated kappa coefficients on the dropout noised test data.

- - 1. Simulated imbalance dataset

In order to get a different proportion (0.1:1, 0.3:1, 0.5:1, 0.7:1, 0.9:1, 1:0.9, 1:0.7, 1:0.5, 1:0.3 and 1:0.1) of the imbalance dataset, we used two cell types (Hepatocyte and GamaDetaT) from the liver data. We first down sampled each cell type to 300 cells. Then, we adjust the proportion of cell numbers in two types to get datasets with different cell proportion. Similarly, we did five-fold cross validation on datasets with different simulated imbalance noise.

- 1. **Cross platform datasets**

The annotation label of different platform datasets from the same tissue is not consistent. So, we unified the labels and remove cell types absent in any of the datasets.

***Liver datasets*:**

*10x Dataset [1]*

We have descripted in section 1.1.

*mCel-seq2 [8]*

| Expression profile | We got the normalized expression profile from the original research. |
| --- | --- |
| Cell type information | We got the cluster information from the original research. We could not find the cell type information with cluster. So, we used the differential expression genes information offered by the original research to overlap with liver marker genes in 10x dataset to get cell types. (overlap the top 20 DE genes in each cluster). We removed clusters with multi-type marker and no marker. |
| Final types | “T_NK”, “Cholangiocytes”, “Macrophage”, “B_cells”, “Hepatocytes”, “Central_venous_LSECs”, “Periportal_LSECs” |

Note: For unified datasets, we merged “AlfaBetaT”, “GamaDetaT” and “NK_like” into “T_NK”, “Inflammatory_Macs” and “Non-inflammatory_Macs” into “Macrophage”, “Plasma_cells” and “Mature_B_cells” into “B_cell”. And we removed the “Portal_endothelial”, “Erythroid_cells” and “Hepatic_Stellate_Cells” types because these types appear in only one dataset.

***Pancreas datasets:***

*Drop-seq Dataset [2]*

We have descripted in section 1.1.

*Smart-seq2 Dataset [9]*

| Expression profile | The resource came from the original research which is count matrix. We used cpm to normalize the count matrix. |
| --- | --- |
| Cell type information | We got this information in the original research. |
| Final types | “acinar”, “alpha”, “beta”, “delta”, “ductal”, “endothelial”, “epsilon”, “gamma”, “mast” |

We removed the “activated_stellate”, “quiescent_stellate”, “schwann” and “macrophage” types because these types appear in only one dataset.

1. **The parameter of filter gene nodes**

In gCAnno, we filtered gene nodes in our weighted cell type-gene bipartite graph (wCGBG) by gene expression abundance. We set the parameter as 30%, which means gene nodes are filtered when their expression abundance less than 30% in all cell types, accounting for calculation efficiency. We evaluated the performance on different parameters (10%, 20%, 30%, 40% and 50%). The results are shown in below table. The parameter was not sensitive between 10% to 30%. For speeding up the calculation efficiency, we selected 30% as a parameter in all of our test. Users can adjust it according to their own time and calculation device. Overall performance evaluation datasets used the average kappa coefficients.

|  | | 10% | 20% | 30% | 40% | 50% |
| --- | --- | --- | --- | --- | --- | --- |
| Liver | kappa | 0.92 | 0.92 | 0.91 | 0.88 | 0.81 |
|  | time | 96.25 | 40.33 | 20.13 | 11.9 | 8.51 |
| Pancreas | kappa | 0.92 | 0.92 | 0.9 | 0.89 | 0.86 |
|  | time | 104.16 | 56.2 | 30.19 | 17.97 | 11.24 |
| AT root | kappa | 0.73 | 0.72 | 0.7 | 0.7 | 0.66 |
|  | time | 282.04 | 136.43 | 75.62 | 45.92 | 29.67 |
| HCC&ICCA | kappa | 0.84 | 0.82 | 0.8 | 0.79 | 0.79 |
|  | time | 54.17 | 21.88 | 10.94 | 6.45 | 4.71 |
| ref:mCel-seq2 query:10x | kappa | 0.93 | 0.94 | 0.94 | 0.93 | 0.93 |
|  | time | 73.74 | 36.09 | 19.4 | 11.87 | 9.26 |
| ref:10x query:mCel-seq2 | kappa | 0.93 | 0.92 | 0.93 | 0.92 | 0.92 |
|  | time | 45.09 | 16.57 | 8.79 | 6.69 | 5.54 |
| ref:drop-seq query:smart-seq2 | kappa | 0.98 | 0.95 | 0.92 | 0.84 | 0.71 |
|  | time | 68.12 | 30.69 | 16.15 | 9.86 | 7.1 |
| ref:smart-seq2 query:drop-seq | kappa | 0.86 | 0.85 | 0.88 | 0.85 | 0.83 |
|  | time | 170.9 | 134.88 | 117.14 | 96.79 | 72.4 |

Note: ref means reference dataset, query means query dataset and kappa means kappa coefficient.

**References**

1. MacParland SA, Liu JC, Ma XZ, Innes BT, Bartczak AM, Gage BK, Manuel J, Khuu N, Echeverri J, Linares I *et al*: **Single cell RNA sequencing of human liver reveals distinct intrahepatic macrophage populations**. *Nat Commun* 2018, **9**(1):4383.

2. Baron M, Veres A, Wolock SL, Faust AL, Gaujoux R, Vetere A, Ryu JH, Wagner BK, Shen-Orr SS, Klein AM *et al*: **A Single-Cell Transcriptomic Map of the Human and Mouse Pancreas Reveals Inter- and Intra-cell Population Structure**. *Cell Syst* 2016, **3**(4):346-360 e344.

3. Ma L, Hernandez MO, Zhao Y, Mehta M, Tran B, Kelly M, Rae Z, Hernandez JM, Davis JL, Martin SP *et al*: **Tumor Cell Biodiversity Drives Microenvironmental Reprogramming in Liver Cancer**. *Cancer Cell* 2019, **36**(4):418-430 e416.

4. Zhang TQ, Xu ZG, Shang GD, Wang JW: **A Single-Cell RNA Sequencing Profiles the Developmental Landscape of Arabidopsis Root**. *Mol Plant* 2019, **12**(5):648-660.

5. Tabula Muris C, Overall c, Logistical c, Organ c, processing, Library p, sequencing, Computational data a, Cell type a, Writing g *et al*: **Single-cell transcriptomics of 20 mouse organs creates a Tabula Muris**. *Nature* 2018, **562**(7727):367-372.

6. Abdelaal T, Michielsen L, Cats D, Hoogduin D, Mei H, Reinders MJT, Mahfouz A: **A comparison of automatic cell identification methods for single-cell RNA sequencing data**. *Genome Biol* 2019, **20**(1):194.

7. Alquicira-Hernandez J, Sathe A, Ji HP, Nguyen Q, Powell JE: **scPred: accurate supervised method for cell-type classification from single-cell RNA-seq data**. *Genome Biol* 2019, **20**(1):264.

8. Aizarani N, Saviano A, Sagar, Mailly L, Durand S, Herman JS, Pessaux P, Baumert TF, Grun D: **A human liver cell atlas reveals heterogeneity and epithelial progenitors**. *Nature* 2019, **572**(7768):199-204.

9. Segerstolpe A, Palasantza A, Eliasson P, Andersson EM, Andreasson AC, Sun X, Picelli S, Sabirsh A, Clausen M, Bjursell MK *et al*: **Single-Cell Transcriptome Profiling of Human Pancreatic Islets in Health and Type 2 Diabetes**. *Cell Metab* 2016, **24**(4):593-607.
