# Supplementary material for: gCAnno: a graph-based single cell type annotation method
Source: BMC Genomics. 2020 Nov 23;21:823. doi: 10.1186/s12864-020-07223-4 (PMC7686723; doi:10.1186/s12864-020-07223-4)

Liver

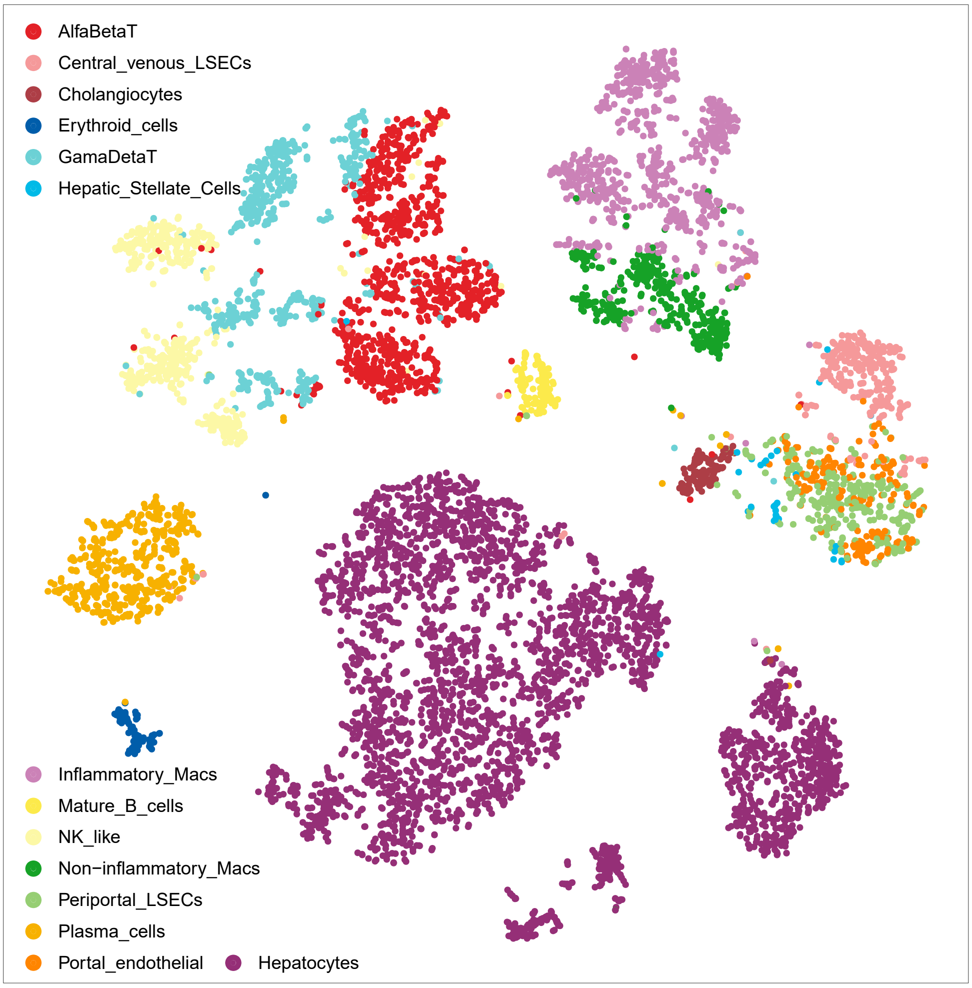
AlfaBetaT
Central_venous_LSECs
Cholangiocytes
Erythroid_cells
GamaDetaT
Hepatic_Stellate_Cells
Inflammatory_Macs
Mature_B_cells
NK_like
Non−inflammatory_Macs
Periportal_LSECs
Plasma_cells
Portal_endothelial
Hepatocytes

Hepatocytes

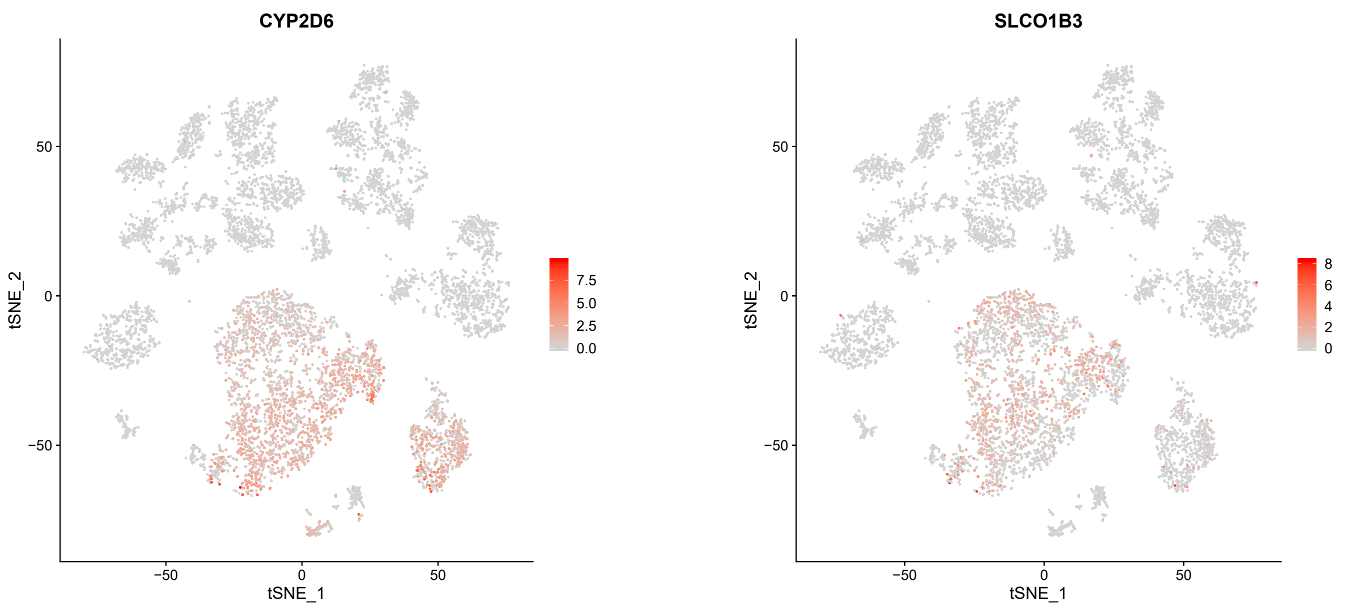

Portal_endothelial

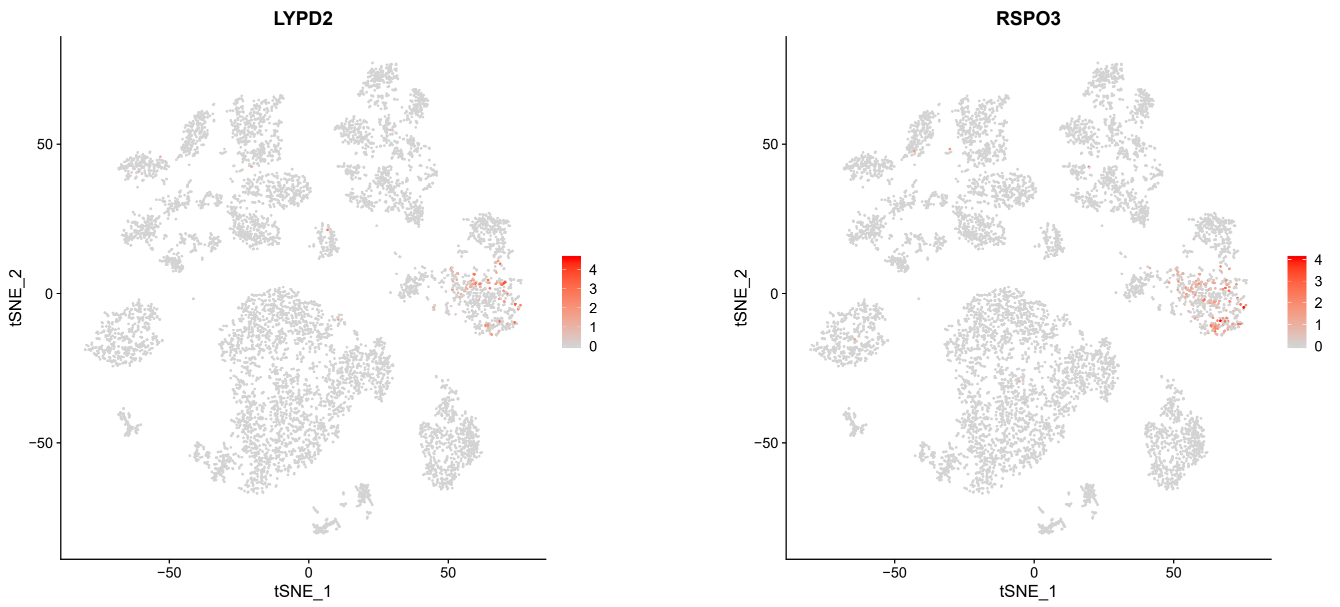

AlfaBetaT

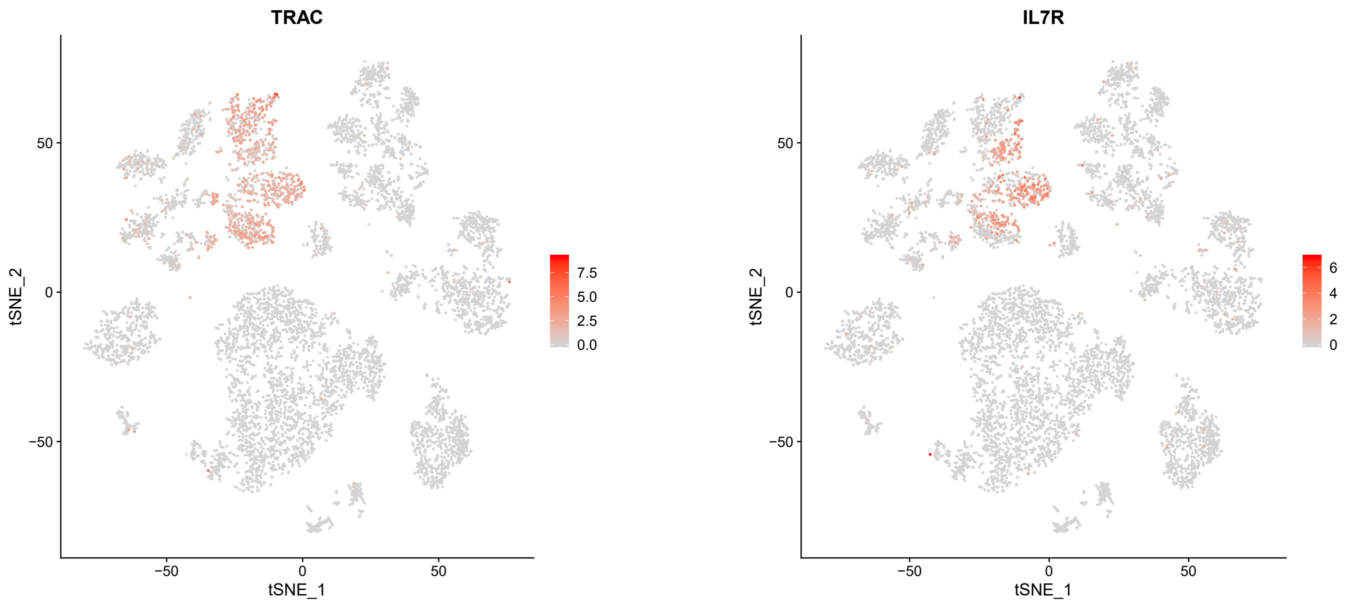

NK_like

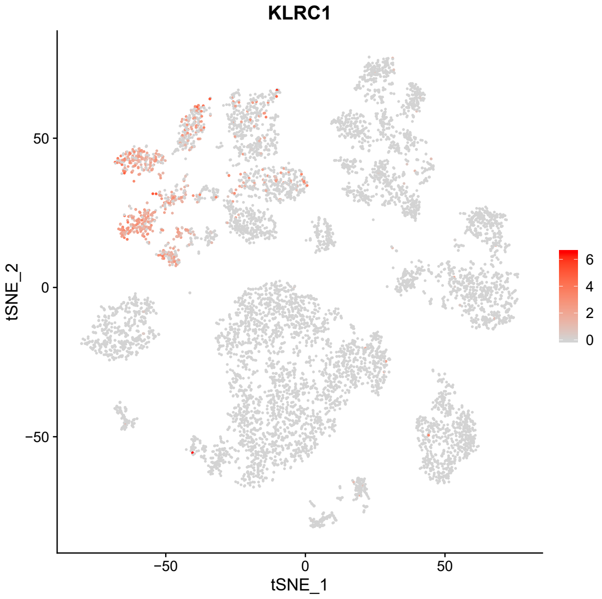

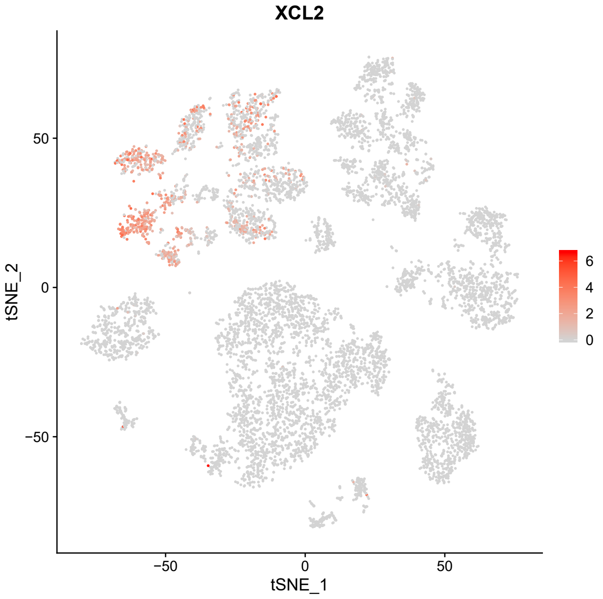

GamaDetaT

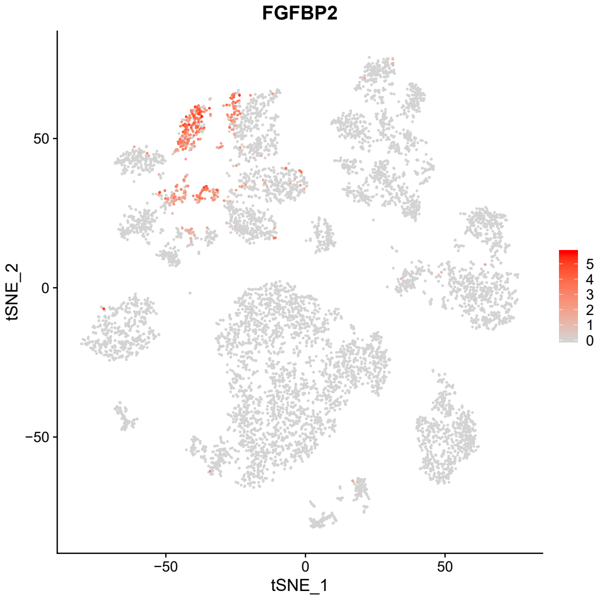

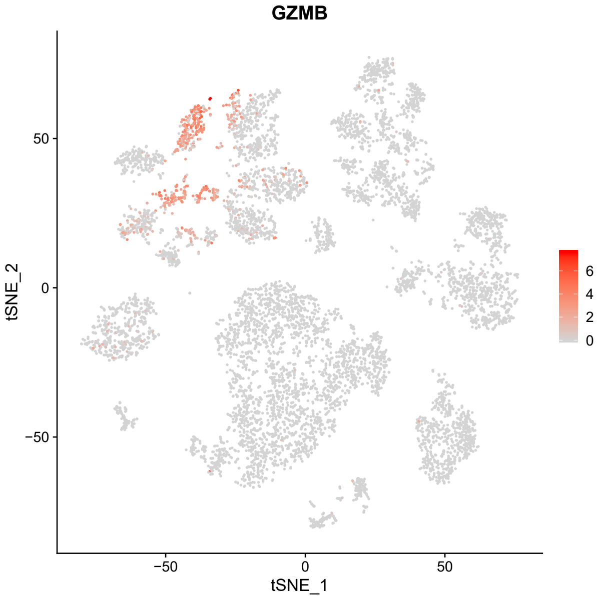

Erythroid_cells

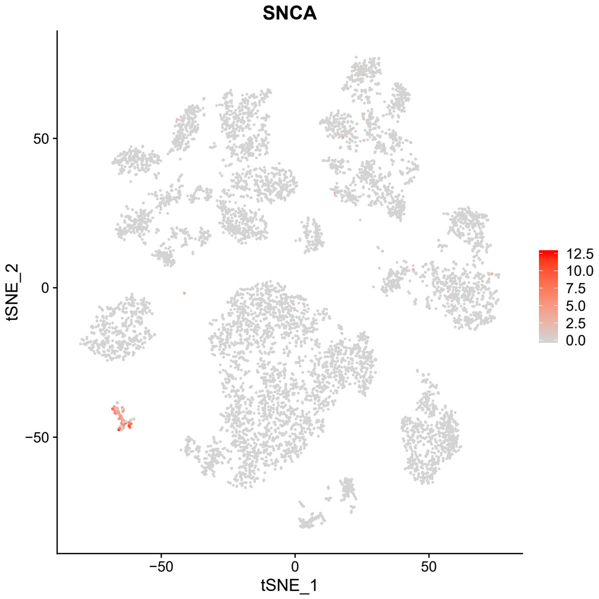

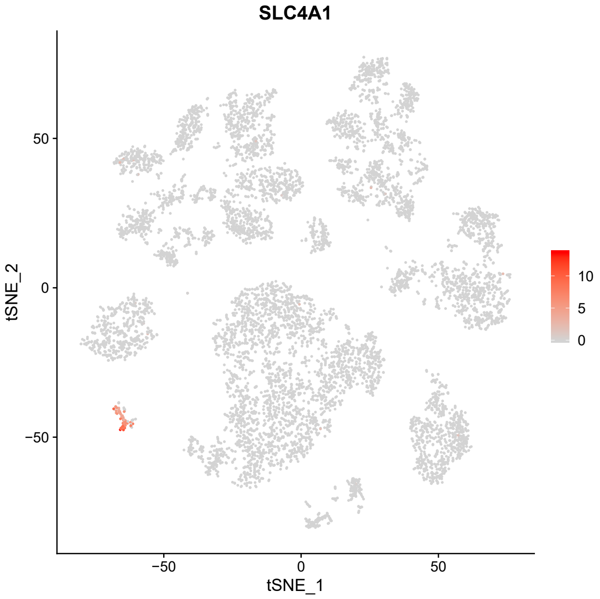

Mature_B_cells

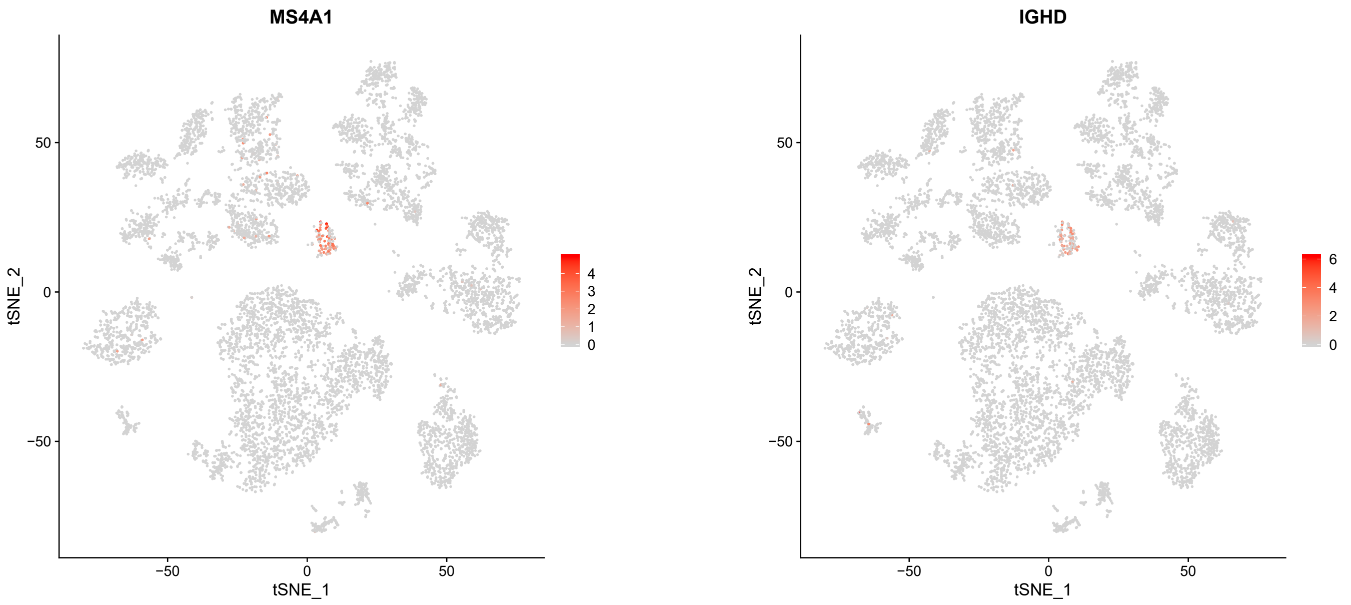

Periportal_LSECs

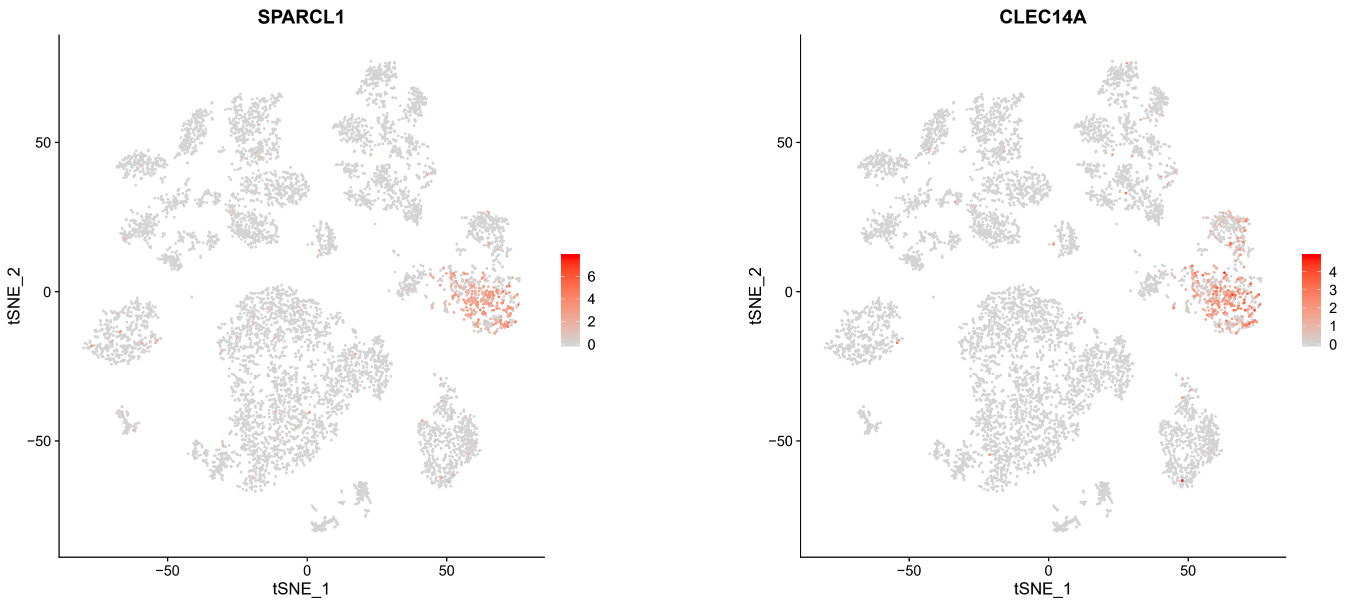

Inflammatory_Macs

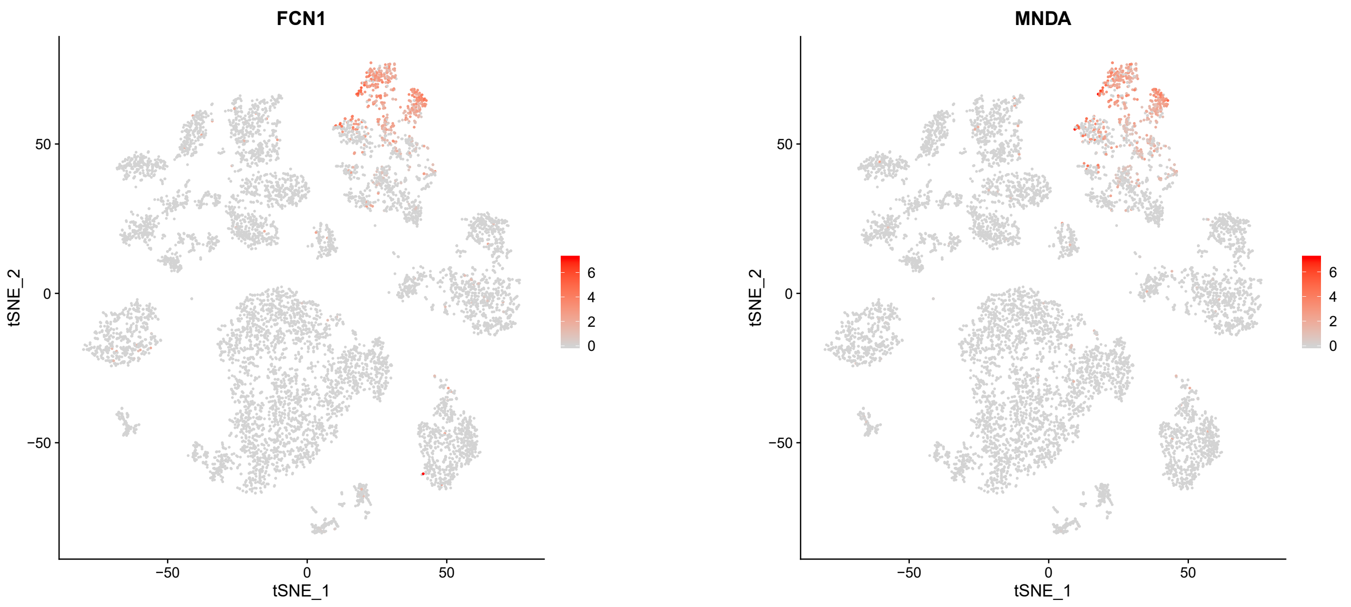

Central_venous_LSECs

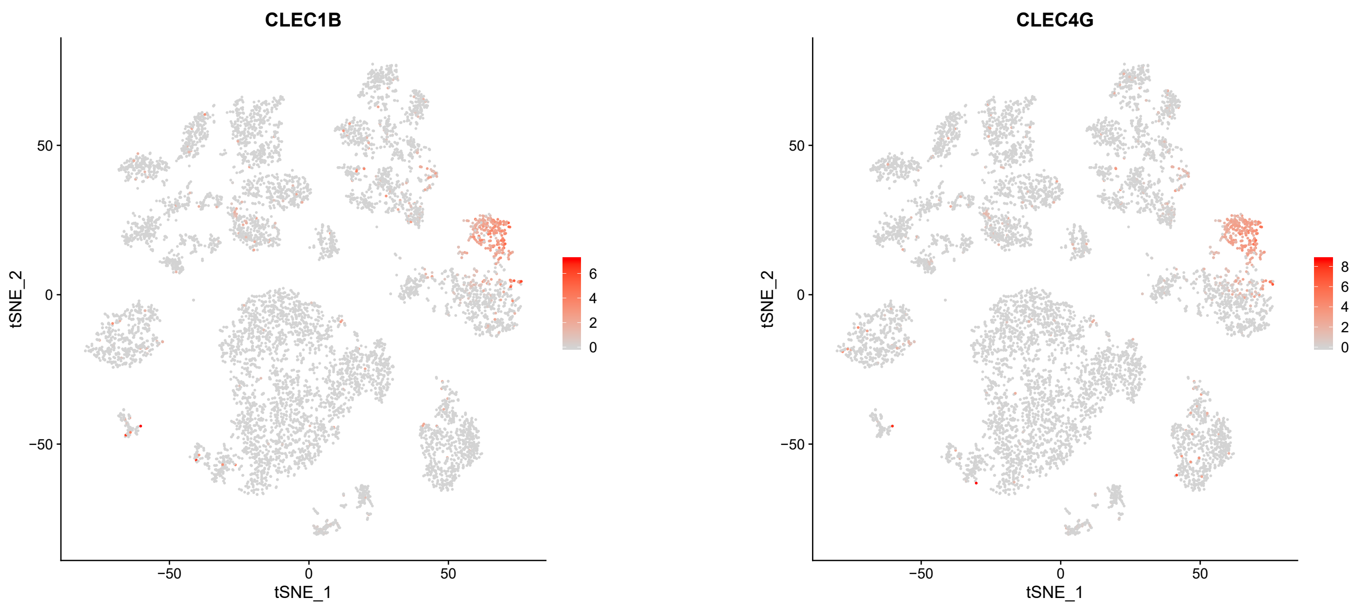

Non-inflammatory_Macs

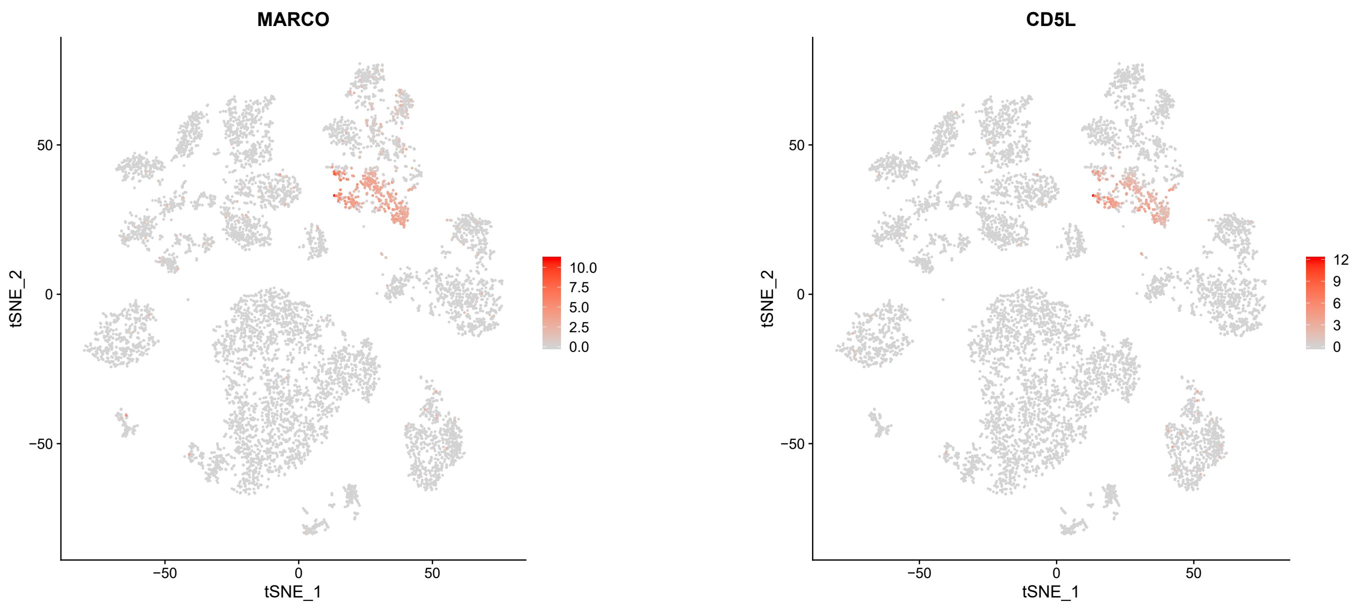

Plasma_cells

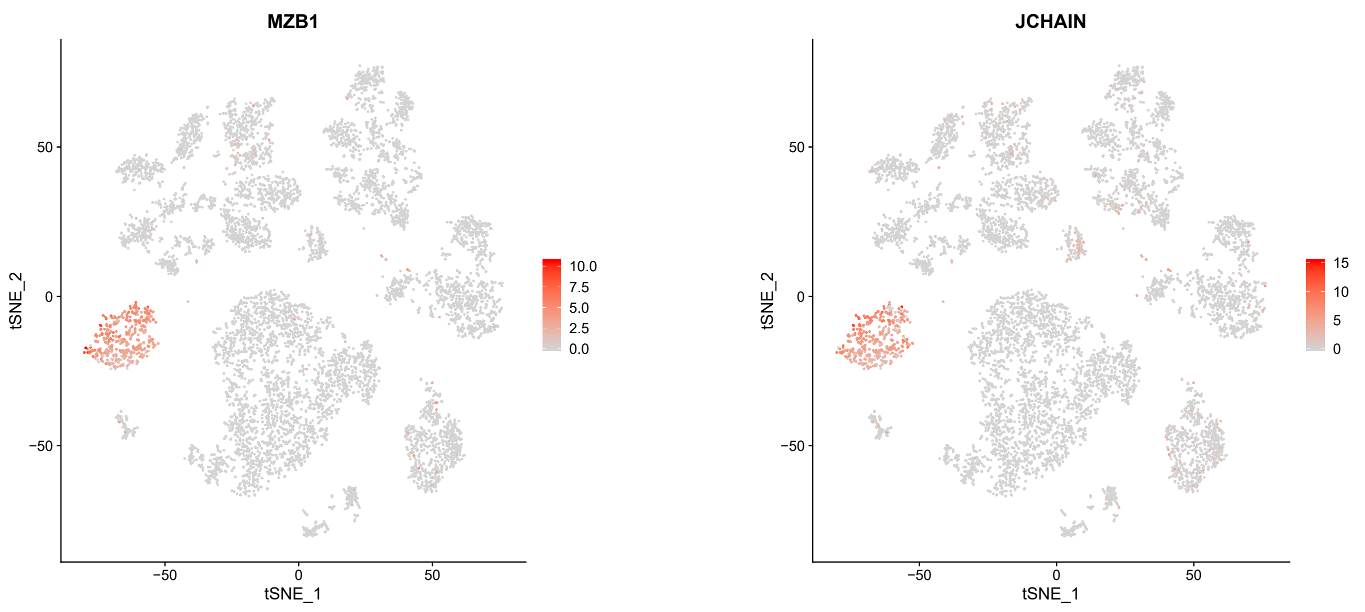

Cholangiocytes

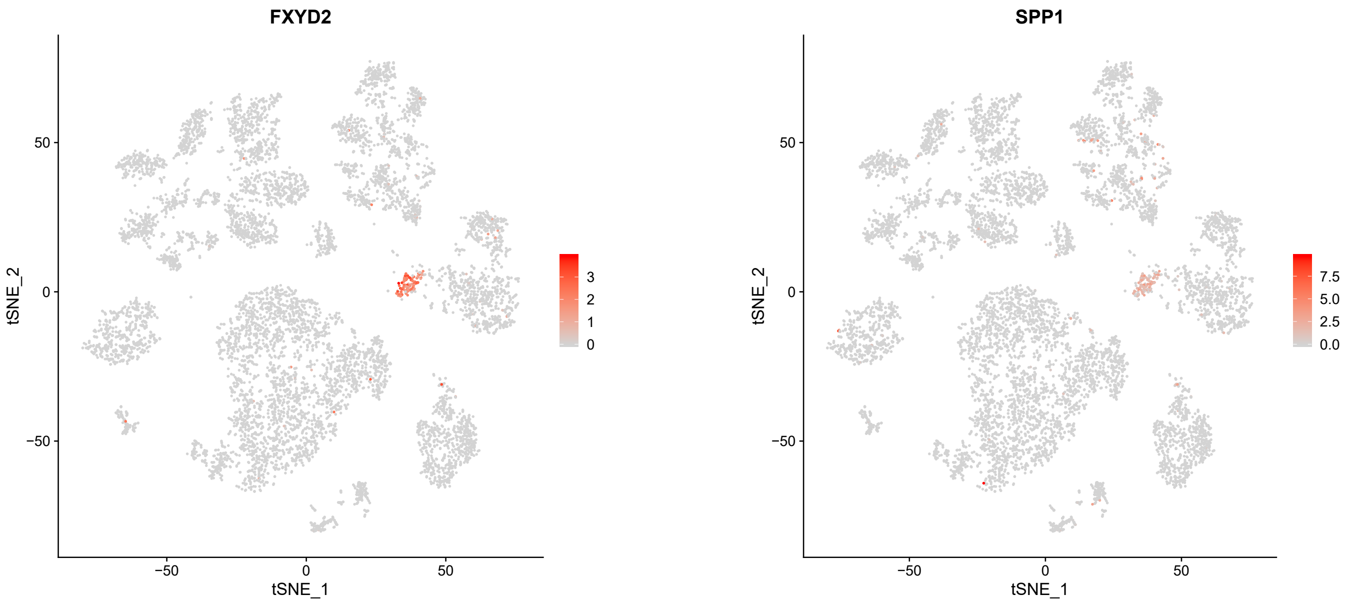

Hepatic_Stellate_Cells

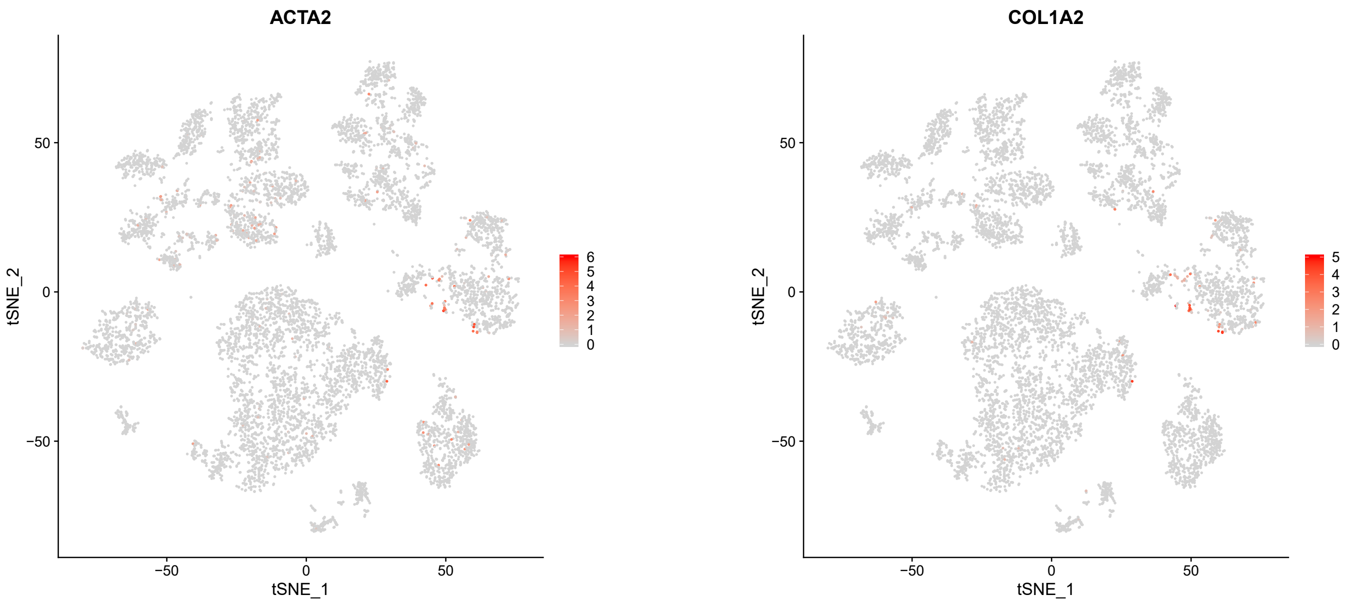

Supplement: Supplementary file 10 — Additional file 10: Figure S6. An example of top 2 specific genes in each cell type of liver dataset. In tSNE plot, each gene specific expressed in corresponding cell type in red color. The shade of color means the expression value. [file 12864_2020_7223_MOESM10_ESM.pdf]
