# Supplementary material for: gCAnno: a graph-based single cell type annotation method
Source: BMC Genomics. 2020 Nov 23;21:823. doi: 10.1186/s12864-020-07223-4 (PMC7686723; doi:10.1186/s12864-020-07223-4)
Supplement: Supplementary file 15 — Additional file 15: Figure S9. Comparisons of gCAnno with Scmap-Cluster, Scmap-Cell, scPred, Chetah and SVM on (a) HCC and ICCA reference dropout dataset, (b) HCC and ICCA query dropout dataset, (c) AT root reference dropout dataset, (d) AT root query dropout dataset and (e) imbalance dataset. [file 12864_2020_7223_MOESM15_ESM.pdf]

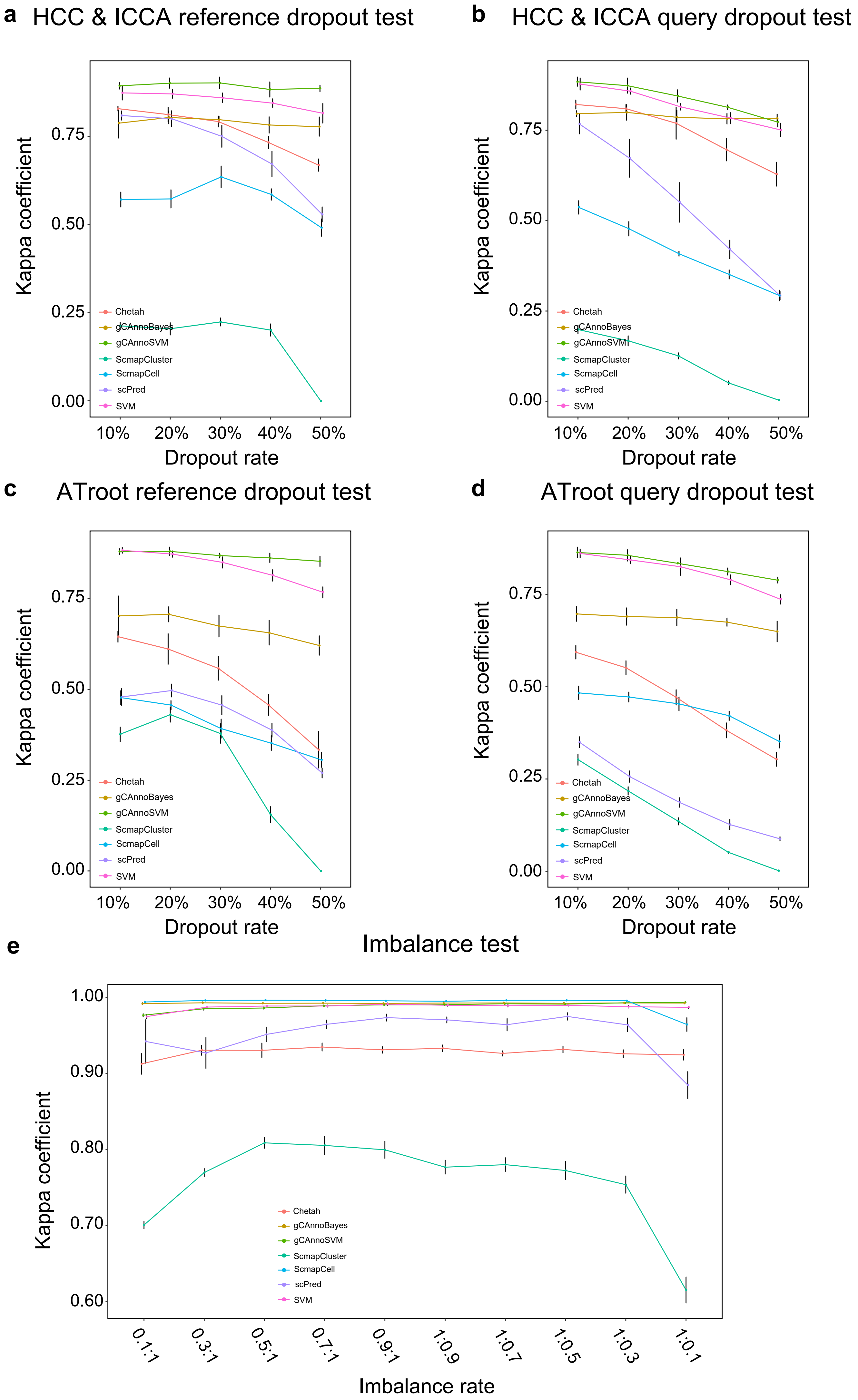

a
HCC & ICCA reference dropout test
b
HCC & ICCA query dropout test
c
ATroot reference dropout test
d
ATroot query dropout test
Kappa coefficient
0.00
0.25
0.50
0.75
Dropout rate
10%
20%
30%
40%
50%
Chetah
gCAnnoBayes
gCAnnoSVM
ScmapCluster
ScmapCell
scPred
SVM
e
Imbalance test
0.60
0.70
0.80
0.90
1.00
Imbalance rate
0.1:1
0.3:1
0.5:1
0.7:1
0.9:1
1:0.9
1:0.7
1:0.5
1:0.3
1:0.1
